# A Dual‐Functional Biohybrid Nanorobot to Synergistically Eradicate Biofilm and Degrade Antibiotic Resistance Genes

**DOI:** 10.1002/advs.75287

**Published:** 2026-04-14

**Authors:** Junzheng Zhang, Tong Dou, Luokai Wang, Jingxian Yang, Wenrui Wang, Shuaitian Guo, Xiangyu Li, Xuefang Huang, Yunlei Xianyu, Dongsheng Wang

**Affiliations:** ^1^ College of Environmental and Resource Sciences Zhejiang University Hangzhou China; ^2^ Innovation Center of Yangtze River Delta Zhejiang University Jiashan China; ^3^ School of Clinical and Basic Medical Sciences Shandong First Medical University & Shandong Academy of Medical Sciences Jinan China; ^4^ Division of Molecular Bacterial Epidemiology & Infectious Diseases Institute of Veterinary Bacteriology University of Bern Bern Switzerland; ^5^ Institute of Biotechnology Zhejiang University Hangzhou China; ^6^ Department of Clinical Laboratory Sir Run Run Shaw Hospital Zhejiang University School of Medicine Hangzhou China; ^7^ College of Biosystems Engineering and Food Science Zhejiang Key Laboratory of Agro‐food Resources and High‐value Utilization Zhejiang University Hangzhou China

**Keywords:** Antibiotic resistance genes, bacteriophages, biofilm disruption, nanorobots, nanozymes

## Abstract

The environmental dissemination of antibiotic‐resistant bacteria and associated resistance genes poses a serious threat to public health and ecological safety, while persistent biofilms serve as reservoirs and transmission hubs for antibiotic resistance genes (ARGs). Current strategies are unable to synergistically eradicate biofilm and degrade ARGs. Here, we developed a dual‐functional biohybrid nanorobot that integrates the lytic bacteriophage (N4) with Pd nanozymes to eradicate multidrug‐resistant *Escherichia coli* NDM‐1 biofilms and degrade released ARGs. Phage N4 enables targeted bacterial lysis and precise delivery of Pd nanozymes, which catalyze the production of reactive oxygen species to enhance antibacterial activity, disrupt biofilm structure, and degrade liberated plasmid‐encoded *bla*_NDM‐1_ genes. This N4@Pd nanorobot achieves over 95% biofilm removal and approximately 2.78 log_10_ reduction in ARGs. Transcriptomic analyses reveal that the nanorobot treatment inhibits core bacterial metabolic pathways and key regulators of biofilm maintenance. In simulated wastewater, the nanorobot maintains over 90% biofilm removal and approximately 2.06 log_10_ reduction in ARGs, underscoring its potential in environmental water treatment. This study presents a promising strategy to counteract the spread of antibiotic resistance through integrated biofilm disruption and genetic decontamination.

## Graphical abstract

A biohybrid nanorobot integrating lytic bacteriophage N4 with Pd nanozymes is developed for targeted eradication of multidrug‐resistant *E. coli* biofilms. Synergistic bacterial lysis and ROS‐mediated oxidation enable simultaneous biofilm removal and antibiotic resistance genes degradation, maintaining high efficacy in complex wastewater environments.

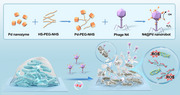

## 1 Introduction

The rapid and widespread dissemination of antibiotic‐resistant bacteria (ARB) and antibiotic resistance genes (ARGs) has emerged as critical challenges threatening both public health and environmental safety [1, 2]. Biofilms, which serve as ecological niches for resistant bacteria and hotspots for horizontal gene transfer, further exacerbate these concerns [3, 4]. In aquatic environments such as wastewater treatment, surface water, and drinking water distribution networks, biofilms act as long‐term reservoirs of ARB and ARGs [5]. These communities are characterized by high cell density, metabolic cooperation, and active genetic exchange, which collectively facilitate the evolution and stabilization of resistance traits [6, 7, 8].

Conventional strategies for biofilm eradication include antibiotic treatment, oxidative disinfectants, and physical disruption, which have limited effectiveness and can result in unintended ecological consequences [9]. Antibiotics exhibit poor penetration through the extracellular polymeric substances (EPS) matrix, resulting in sub‐lethal exposure that facilitates resistance evolution [10]. Chemical disinfectants such as chlorine or ozone can inactivate microorganisms but frequently produce harmful disinfection by‐products, posing concerns for both the environment and human health [11]. Mechanical methods such as ultrasonication may detach biofilms from surfaces but often leave behind residual ARGs that remain environmentally mobile [12]. Hydrogen peroxide (H_2_O_2_)‐based advanced oxidation processes (AOPs) are widely employed for pollutant degradation and microbial inactivation [13, 14, 15, 16]. Compared with sodium hypochlorite disinfection, H_2_O_2_‐based processes generate significantly fewer halogenated disinfection by‐products due to the absence of reactive chlorine species [17, 18]. Typical H_2_O_2_ dosing concentrations range from 13 mg·L^−1^ to 30 g·L^−1^, with lower doses (13–75 mg·L^−1^) for municipal secondary effluent and higher doses (1.5–30 g·L^−1^) for recalcitrant industrial wastewater or landfill leachate [19, 20, 21, 22, 23]. However, high H_2_O_2_ concentrations exhibit strong oxidative and corrosive properties, posing risks to material stability and operational safety [24, 25]. Moreover, conventional AOPs generally lack biological specificity toward resistant pathogens and exhibit limited capability in degrading extracellular genetic material, particularly ARGs released during bacterial lysis [26]. These limitations underscore the necessity of developing antimicrobial approaches that can concurrently eradicate biofilms and degrade ARGs, thereby preventing genetic pollution and reducing the risk of resistance rebound.

Intelligent nanotechnology provides a powerful and transformative platform to address the growing challenge of antibiotic resistance [27, 28]. Among emerging approaches, antimicrobial nanorobots have gained increasing attention as active therapeutic agents capable of targeting persistent multidrug‐resistant bacterial biofilms and their associated resistance genes [29]. Antimicrobial nanorobots can be engineered with surface chemistry and catalytic propulsion that enable autonomous motion, allowing deep penetration into biofilm matrices and localized antimicrobial action [30]. Their self‐propelling behavior facilitates navigation through complex microenvironments, enhancing contact with bacterial cells and improving treatment efficacy [31]. In addition, their capacity to deliver catalytic agents or therapeutic biomolecules supports multimodal attack mechanisms that disrupt EPS integrity, eliminate embedded bacteria, and prevent biofilm re‐establishment [32, 33]. For example, catalytically active iron oxide nanoparticles have been integrated into antimicrobial nanorobots capable of precise motion control and bactericidal reactive oxygen species (ROS) generation while simultaneously degrading the EPS matrix [34]. Despite these advances, the inactivation of ARB unavoidably releases ARGs, which remain environmentally persistent and pose substantial ecological risks. This unresolved issue highlights the need for nanorobots that can concurrently eliminate ARB and degrade their liberated ARGs to curb resistome dissemination.

Bacteriophages (phages), as natural predators of bacteria, are promising for targeted microbial control due to their inherent ability to specifically recognize and lyse bacterial hosts [35, 36]. The engineered integration of phages with functional nanomaterials can enhance the antibacterial performance [37, 38], which can be viewed as nanorobots with recognition and sterilization capacity. Among these functional materials, nanozymes are particularly attractive because they exhibit high catalytic efficiency, tunable activity, and resistance to harsh environmental conditions [39, 40]. As a representative example, palladium (Pd) nanozymes exhibit robust peroxidase‐like activity that can be activated in the acidic biofilm microenvironment [41, 42], potentially facilitating the oxidative degradation of released ARGs and serving as a potent catalytic “weapon” module in nanorobots. The integration of phages and nanozymes into biohybrid nanorobots offers a platform to address the dual challenge of eliminating multidrug‐resistant biofilms and mitigating the environmental dissemination of ARGs.

**SCHEME 1:**
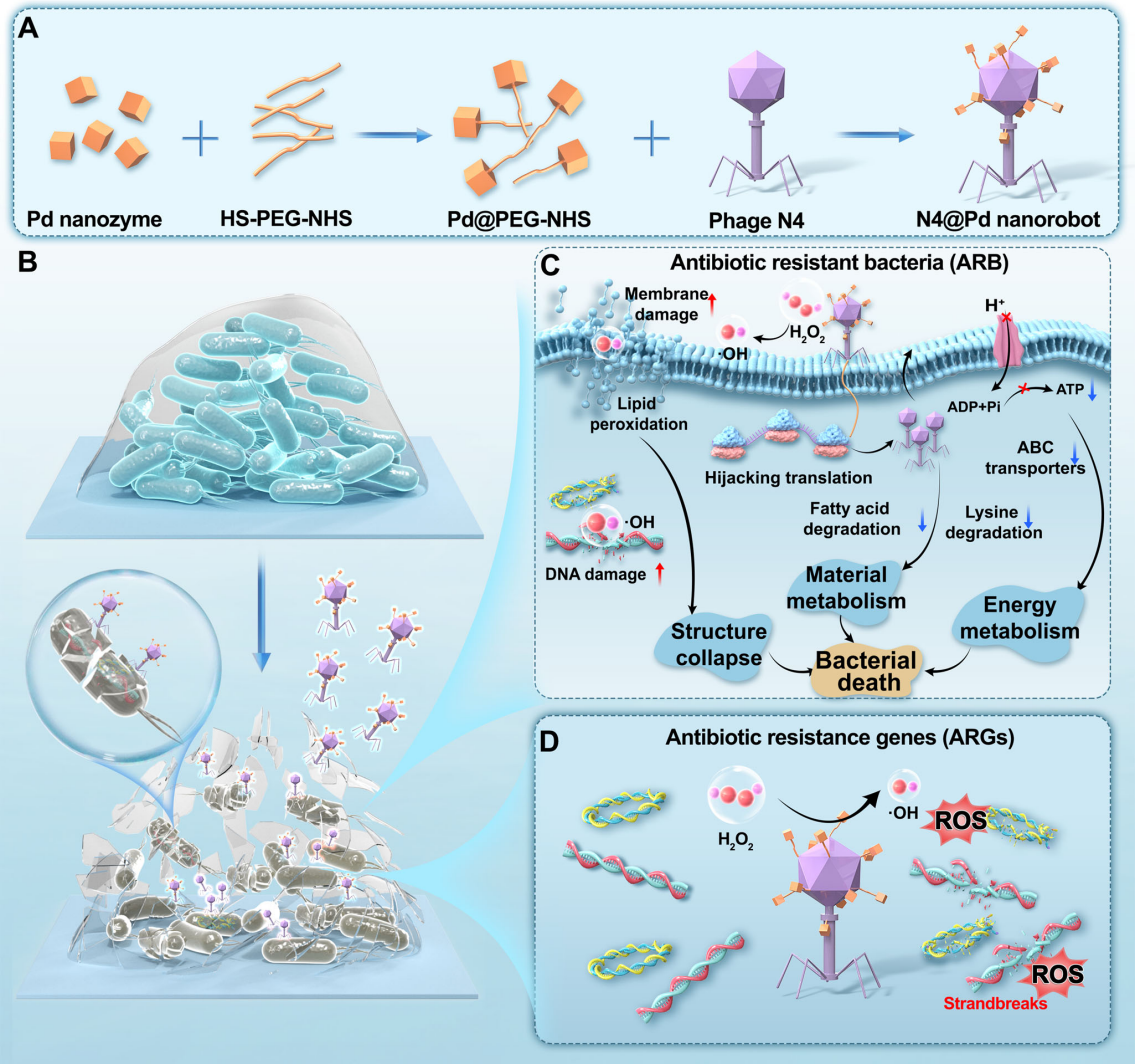
Schematic illustration of the N4@Pd nanorobot for synergistic elimination of *E. coli* NDM‐1 biofilms and degradation of ARGs. (A) Modular assembly of Pd nanozymes onto phage N4. (B) Antibiofilm mechanism of the N4@Pd nanorobot. (C) Synergistic antibacterial action combining phage lysis and ROS‐enhanced attack. (D) Hydroxyl radicals (·OH) driven oxidative fragmentation of ARGs, enabling post‐killing genetic clearance.

In this study, we have engineered a dual‐functional N4@Pd nanorobot that integrates phage‐mediated bacterial lysis with nanozyme‐catalyzed oxidative activity (Scheme 1). This nanorobot incorporates a lytic phage N4, exhibiting high specificity toward *Escherichia coli* NDM‐1. To address the challenge of residual ARGs release after phage lysis, Pd nanozymes were decorated onto the N4 capsid, enabling ROS generation that strengthens bactericidal potency while simultaneously degrading ARGs, thereby minimizing the risk of horizontal gene transfer. Through this cascade antimicrobial mechanism, the N4@Pd nanorobot achieves deep biofilm penetration, precise elimination of ARB, and simultaneous degradation of ARGs. This synergistic strategy establishes a transformative platform that not only ensures biofilm clearance but also effectively mitigates the genetic risks associated with antibiotic resistance dissemination, offering a sustainable and environmentally responsible solution for water treatment and microbial control.

## 2 Results and Discussion

### 2.1 Characterization of Phage N4 and Pd Nanozymes

**FIGURE 1:**
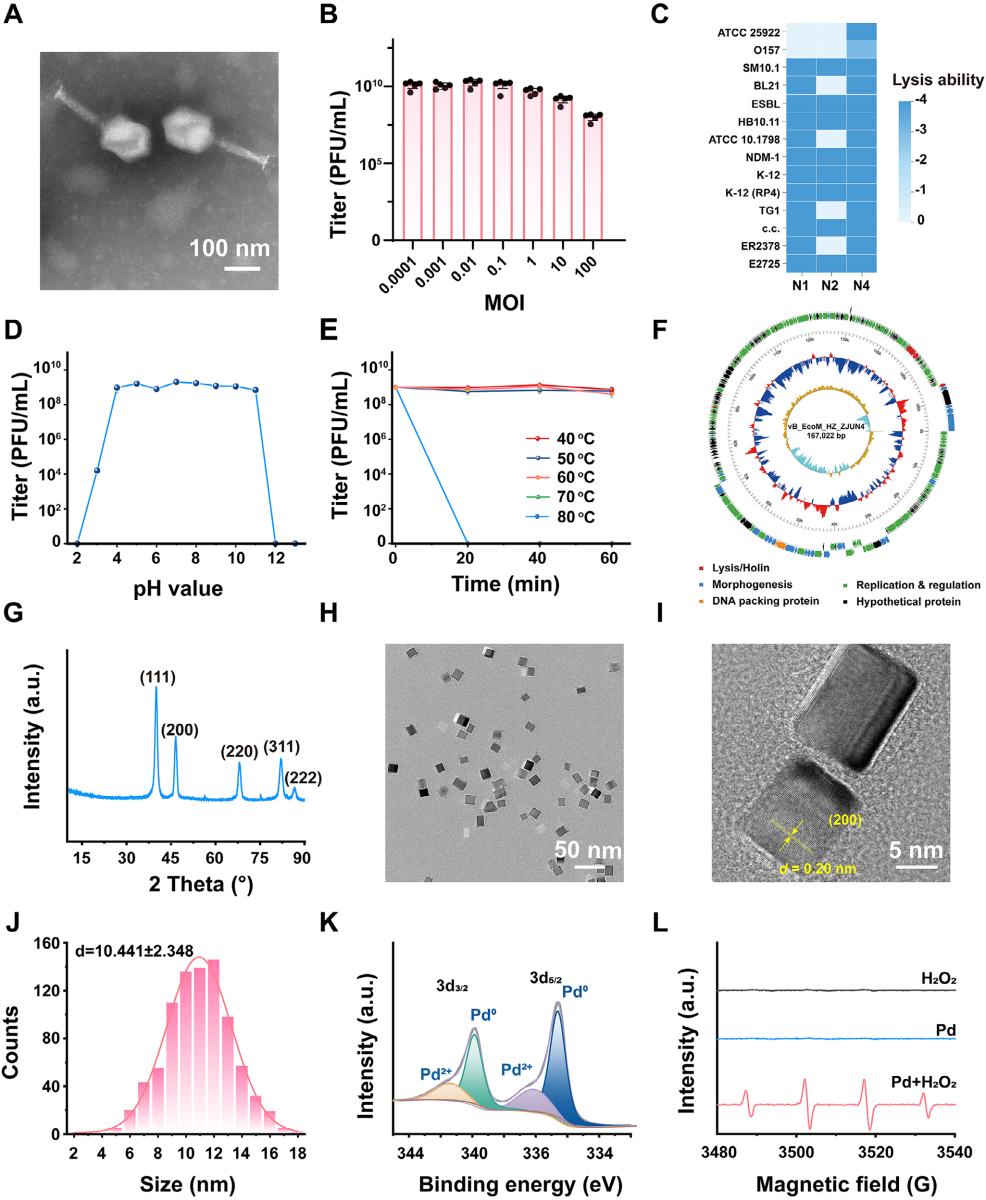
Characterization of bacteriophage N4 and Pd nanozyme. (A) TEM image of phage N4. Scale bar = 100 nm. (B) Titer of phage N4 at different MOI on *E. coli* NDM‐1. (C) Host range of phage N4 against different *E. coli* strains, with phages N1 and N2 (co‐isolated from the same wastewater sample) for comparison. Lytic ability was graded based on plaque clarity and reduction in bacterial lawn density (detailed criteria in Section S1.2). (D) Stability of phage N4 under different pH conditions. (E) Stability of phage N4 under different temperatures. (F) Genome map of phage N4 with functional annotations. (G) XRD pattern of Pd nanozymes. (H) TEM image of Pd nanozymes. Scale bar = 50 nm. (I) HRTEM image of Pd nanozymes. Scale bar = 5 nm. (J) Size distribution of Pd nanozymes. (K) XPS spectrum of Pd nanozymes. (L) ESR spectra of H_2_O_2_ (1 mM), Pd (50 µg·mL^−1^), and Pd (50 µg·mL^−1^) + H_2_O_2_ (1 mM).

Bacteriophage N4 was isolated from municipal wastewater using *E. coli* NDM‐1 as the enrichment host. Transmission electron microscopic (TEM) images showed that phage N4 exhibits an icosahedral capsid coupled with a contractile tail structure, consistent with morphological characteristics associated with myovirus‐like members within the class *Caudoviricetes* (Figure 1A). N4 replicated efficiently on *E. coli* NDM‐1, producing similar final titers across multiplicity of infection (MOI) from 0.0001 to 0.1, with reduced yields only at higher MOI. Under an MOI of 0.01, N4 achieved a high final titer of approximately 1.93 × 10^10^ PFU·mL^−1^ on *E. coli* NDM‐1 (Figure 1B), and its one‐step growth curve revealed a brief latent phase of about 10–20 min, followed by a productive lytic cycle yielding roughly 85 progeny particles per infected cell (Figure S1). N4 showed a broad host range, infecting a wide panel of *E. coli* strains, including multidrug‐resistant clinical isolates as well as non‐resistant laboratory strains (Figure 1C). Among the three phages isolated, N4 exhibited the broadest infectivity profile, which motivated its selection as the biological recognition module of the nanorobot.

In addition to its biological performance, N4 proved highly stable, retaining infectivity across a pH range of 4–11 (Figure 1D) and at temperatures between 20°C and 70°C (Figure 1E) for at least 1 h. Comprehensive genomic mapping showed that N4 possesses a 167 kb genome free of antibiotic and virulence genes (Figure 1F). Phylogenetic analysis confirmed high sequence similarity to previously reported *Escherichia* phages within *Caudoviricetes* (Figure S2), supporting its genetic reliability and traceability. This combination of environmental robustness and genetic safety underscores the suitability of N4 for applications in natural and engineered aquatic systems.

X‐ray diffraction (XRD) patterns of the Pd nanozyme exhibited distinct characteristic peaks at 40.1°, 46.6° and 68.1°, corresponding to the (111), (200) and (220) lattice planes of face‐centered cubic (fcc) Pd (Figure 1G) [43]. TEM images showed that the Pd nanozyme exhibited a cubic morphology with uniform particle sizes and negligible aggregation, indicating high crystallinity and structural integrity (Figure 1H). High‐resolution TEM further revealed periodic lattice fringes across the Pd nanozyme, with an interplanar spacing of approximately 0.20 nm, corresponding to the (200) plane of fcc Pd (Figure 1I). The densely packed lattice fringes further confirmed the high crystallinity of the Pd nanozyme and the predominant exposure of the catalytically active {100} facets. Statistical analysis of TEM images showed uniform cubic morphology with a mean particle size of 10.4 ± 2.3 nm (Figure 1J). High‐angle annular dark‐field TEM and the corresponding elemental mapping (Figure S3A,B) demonstrated a homogeneous distribution of Pd throughout the nanozyme. X‐ray photoelectron spectroscopy (XPS) analysis identified the presence of Pd^0^ and Pd^2+^ states, indicating the coexistence of metallic and oxidized Pd on the nanoparticle surface (Figure 1K). This mixed‐valence state is favorable for catalytic reactions, as it allows for efficient electron transfer and substrate activation.

The peroxidase‐like activity of the Pd nanozyme was evaluated using 3,3’,5,5’‐tetramethylbenzidine (TMB) as a chromogenic probe (absorbance at 652 nm upon oxidation). Ultraviolet‐visible (UV‐vis) spectroscopy showed a time‐dependent increase in TMB oxidation catalyzed by Pd nanocrystals (Figure S4). This catalytic behavior is likely associated with the ability of Pd nanocrystals to activate hydrogen peroxide, leading to the formation of highly reactive hydroxyl radicals (·OH) that drive substrate oxidation. The involvement of ·OH generation during the catalytic process was further examined by electron spin resonance (ESR) spectroscopy. A pronounced ·OH species signal was observed exclusively in the reaction containing both H_2_O_2_ and Pd nanozyme (Figure 1L). This result provides direct evidence that ·OH originates from the Pd‐mediated H_2_O_2_ decomposition, confirming the nanozyme‐driven oxidative pathway. Catalytic kinetics of the Pd nanocrystals followed Michaelis‐Menten behavior, yielding a *V_max_* of 4.69×10^−5^ M·min^−1^ and *K_m_* of 0.45 mM for H_2_O_2_ (Figure S5), indicating moderate substrate affinity and high catalytic efficiency. Collectively, these findings confirm that the Pd nanozyme possesses well‐defined morphology, uniform elemental distribution, and excellent catalytic activity, making it suitable for constructing the biohybrid nanorobot.

### 2.2 Construction and Structural Validation of N4@Pd Nanorobot

**FIGURE 2:**
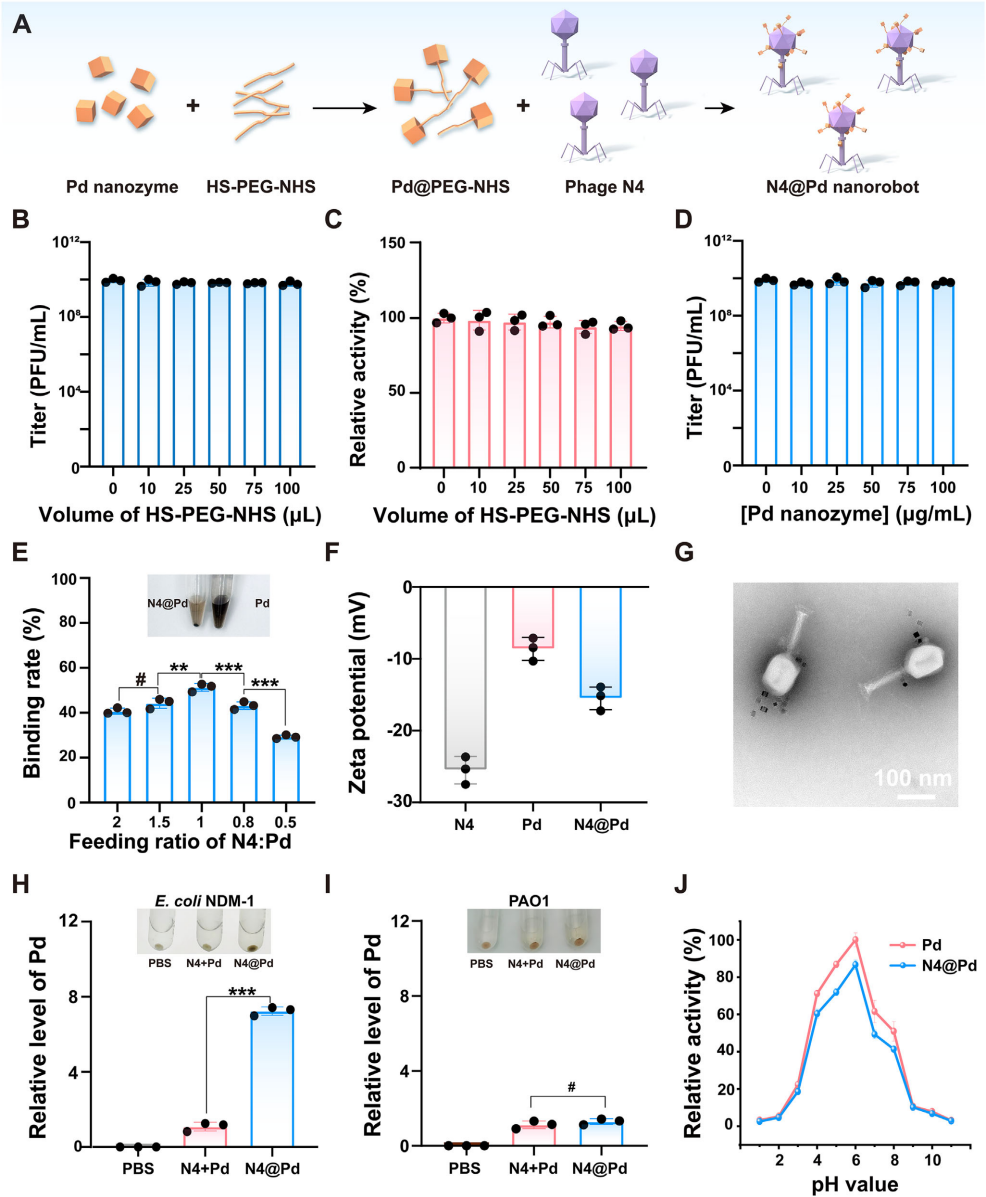
Assembly of N4@Pd nanorobot. (A) Schematic illustration of the assembly of N4@Pd nanorobot. (B) Effect of HS‐PEG‐NHS on N4 infectivity (n = 3). (C) Effect of HS‐PEG‐NHS on the peroxidase‐like activity of Pd nanozymes (n = 3). (D) Effect of Pd nanozyme on N4 infectivity (n = 3). (E) Binding rate of different feeding ratios of N4: Pd (n = 3). (F) Zeta potential of N4, Pd nanozyme, and N4@Pd nanorobot. (G) TEM image of N4@Pd nanorobot. Scale bar = 100 nm. (H) Adsorption of N4@Pd on *E. coli* NDM‐1 (n = 3). (I) Adsorption of N4@Pd on PAO1 (n = 3). (J) Relative peroxidase‐like activity of Pd and N4@Pd (n = 3). Data are presented as mean ± SD. Group differences were analyzed by one‐way ANOVA followed by Tukey's HSD post‐hoc test. Significance is denoted as *p < 0.05, **p < 0.01, ***p < 0.001, and # indicates no significant difference (*P* ≥ 0.05).

HS‐PEG‐NHS was employed as a bifunctional linker to couple the Pd nanozyme with bacteriophage N4, generating the N4@Pd nanorobot (Figure 2A) [44]. The thiol group of HS‐PEG‐NHS forms strong Pd‐S coordination bonds on the Pd nanoparticle surface, while the NHS ester reacts with amino residues on the phage capsid proteins to form stable amide linkages. To ensure that crosslinking did not compromise biological and catalytic performance, a series of HS‐PEG‐NHS concentrations was evaluated. Phage infectivity and the peroxidase‐like activity of Pd remained unchanged (Figure 2B,C), indicating that HS‐PEG‐NHS provides a biocompatible linkage that preserves both viral and catalytic functions. Furthermore, to exclude the possibility of direct interference by Pd nanozyme, N4 infectivity was assessed in the presence of Pd nanozymes alone (Figure 2D). The negligible influence of Pd on phage viability further confirmed the compatibility between the nanozyme and phage components. The feeding ratio between N4 and Pd was optimized to maximize conjugation efficiency. A 1:1 N4/Pd ratio achieved the highest coupling efficiency (∼55.0%) while minimizing steric hindrance (Figure 2E). The zeta potential of native phage N4 was −25.3 ± 2.7 mV, reflecting the abundant negatively charged capsid proteins and phosphate groups (Figure 2F) [45]. In contrast, Pd nanozyme displayed a weakly negative potential (−8.4 ± 1.9 mV) due to PEG surface passivation. After conjugation, the zeta potential increased to −15.1 ± 2.1 mV, indicating partial charge shielding and the deposition of Pd nanozyme on the N4 capsid. TEM images confirmed the formation of the hybrid nanostructure, with multiple Pd nanozymes distributed on the capsid surface of bacteriophage N4 (Figure 2G). Further statistical analysis of 30 representative TEM images (Figure S6) showed that each phage particle carried an average of approximately 6 Pd nanozymes, providing quantitative support for successful phage‐nanozyme conjugation rather than incidental co‐localization. Dynamic light scattering (DLS) analysis was further performed to evaluate the change in hydrodynamic size after conjugation. Pd nanozymes exhibited an average hydrodynamic diameter of 29.8 ± 6.2 nm (PDI = 0.116), whereas native phage N4 showed a larger hydrodynamic diameter of 83.6 ± 14.7 nm (PDI = 0.080). After conjugation, the N4@Pd nanorobot displayed an increased average hydrodynamic diameter of 96.4 ± 18.2 nm (PDI = 0.106), accompanied by a slight broadening of the size distribution (Figure S7). The increase in hydrodynamic size compared with native phage N4 confirmed the attachment of Pd nanozymes onto the phage capsid surface. The relatively narrow size distribution further suggested favorable colloidal stability after conjugation.

Beyond structural confirmation, we investigated whether the nanorobot retained host‐specific targeting by examining the bacterial recognition and binding ability. In phage adsorption assays with *E. coli* NDM‐1, N4@Pd nanorobot showed approximately sixfold higher bacterial aggregation efficiency compared with the physical mixture of N4 and Pd (Figure 2H). The appearance of dense dark aggregates, in contrast to the light precipitate seen with bacteria alone, indicated strong and specific binding of N4@Pd to the target cells. As a non‐host control, *Pseudomonas aeruginosa* PAO1 displayed no significant color change or aggregation (Figure 2I), further supporting the host specificity of the N4@Pd nanorobot and underscoring N4 as a precise navigation component for targeted delivery. Enzymatic assays further showed that the peroxidase‐like activity of Pd was maintained after conjugation, with maximal ROS generation occurring at pH 6.0, approximating the acidic microenvironment of biofilms (Figure 2J). This pH responsiveness implies that catalytic output intensifies within biofilm matrices rather than in neutral bulk water, reducing non‐specific oxidation and enhancing treatment efficiency. These findings confirm that the N4@Pd nanorobot integrates biological recognition (phages) with catalytic ROS generation (Pd) without functional loss, achieving targeted localization, high catalytic accessibility at biofilm matrices, and environmentally adaptive disinfection capability.

Importantly, compared with traditional antibacterial approaches, the N4@Pd nanorobot functions as an intelligent biohybrid rather than a passive catalyst. Conventional nanomaterials, such as Fe_3_O_4_, CeO_2,_ and Cu‐based catalysts, are capable of producing ROS to damage bacterial cells and biofilm matrices, but lack host specificity and are prone to deactivation or non‐productive ROS consumption in complex environmental matrices [46]. Conversely, bacteriophages offer precise host recognition and effective bacterial lysis but face limitations in deep penetration and are prone to resistance development, leading to incomplete removal [47, 48]. The N4@Pd nanorobot overcomes these issues by covalently linking Pd nanozymes to the phage capsid, enabling self‐guided navigation and localized catalytic amplification. This architecture allows the nanorobots to act as a “biological micromachine” capable of self‐guided targeting and local catalytic amplification, thereby surpassing the limitations of conventional passive materials.

### 2.3 ARGs Degradation and Bacterial Eradication

**FIGURE 3:**
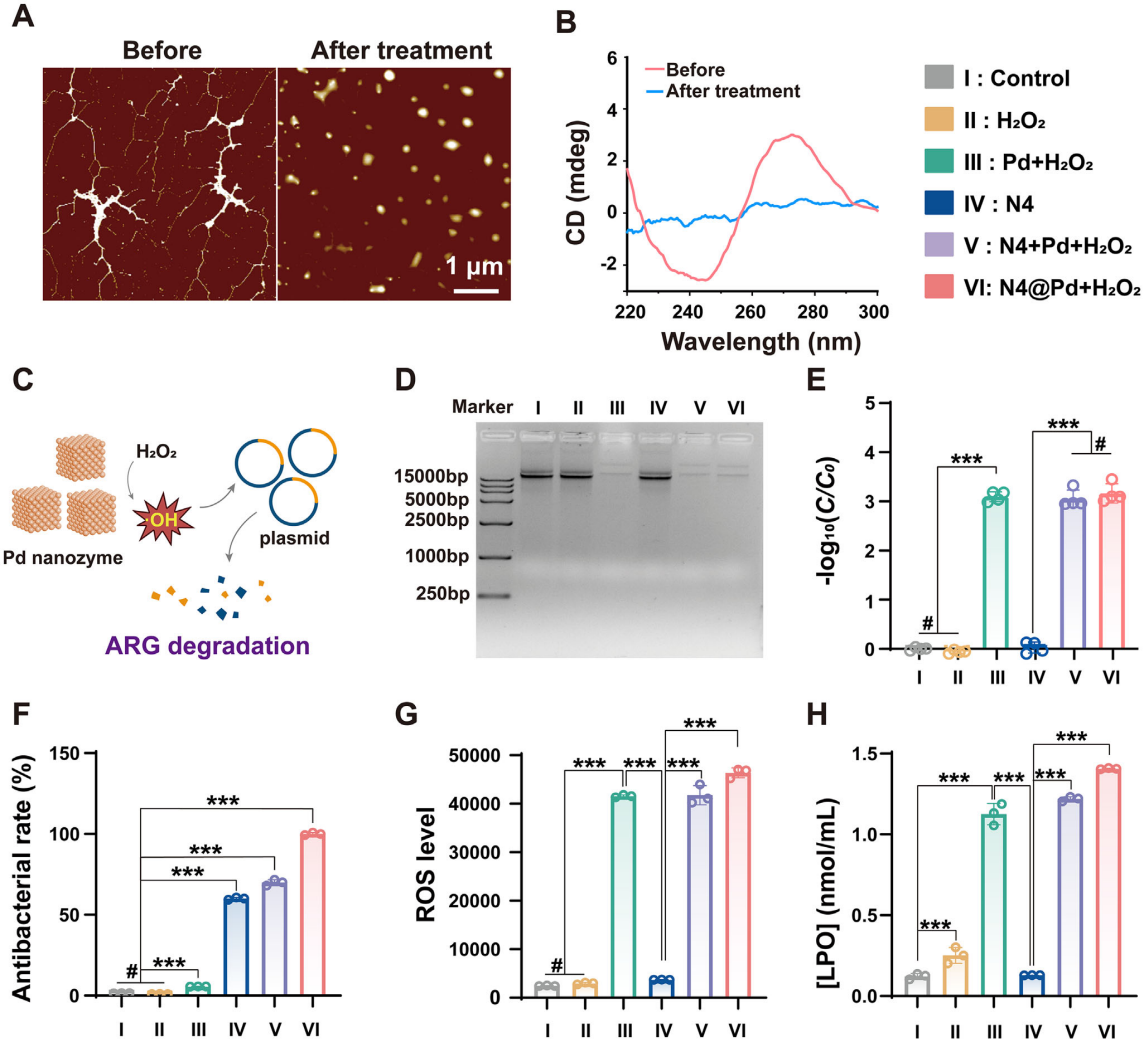
Assessment of ARGs degradation and antibacterial activity of N4@Pd nanorobot. (A) AFM images showing morphological changes of plasmid DNA before and after Pd (50 µg·mL^−1^ Pd) + H_2_O_2_ (1 mM) treatment for 6 h. Scale bar = 1 µm. (B) Circular dichroism spectra of plasmid DNA before and after Pd (50 µg·mL^−1^ Pd) + H_2_O_2_ (1 mM) treatment for 6 h. (C) Schematic illustration of ARGs degradation induced by ·OH. (D) Agarose gel image of plasmid ARGs degradation under different treatments. (E) Quantitative analysis of ARGs copy number reduction under the same condition (n = 4). (F) Antibacterial activity of different treatments against *E. coli* NDM‐1 for 6 h (n = 3). (G) ROS levels in *E. coli* NDM‐1 under different treatments (n = 3). (H) LPO levels in *E. coli* NDM‐1 under different treatments (n = 3). Treatment groups: (I) PBS (control), (II) H_2_O_2_ (1 mM), (III) Pd (50 µg·mL^−1^ for DNA assays; 50 ng·mL^−1^ for cellular assays) + H_2_O_2_ (1 mM), (IV) N4 (1×10^10^ PFU·mL^−1^ for DNA assays; 1×10^7^ PFU·mL^−1^ for cellular assays), (V) Pd + N4 + H_2_O_2_ (same concentrations as above) and (VI) N4@Pd (containing the corresponding N4 and Pd doses) + H_2_O_2_ (1 mM). Data are presented as mean ± SD. Group differences were analyzed by one‐way ANOVA followed by Tukey's HSD post‐hoc test. Significance is denoted as *p < 0.05, **p < 0.01, ***p < 0.001, and # indicates no significant difference (*P* ≥ 0.05).

Given the strong redox activity of Pd nanozymes and their capacity to generate ROS, we next examined whether the Pd component of the N4@Pd nanorobot could mediate ROS‐dependent degradation of plasmid DNA. Atomic force microscopy (AFM) revealed that untreated plasmid DNA displayed continuous, smooth fibrous strands characteristic of intact double‐stranded structures (Figure 3A; Figure S8). Notably, plasmid DNA treated with H_2_O_2_ (1 mM) alone exhibited negligible morphological changes and largely retained the continuous fibrous network, indicating limited DNA degradation (Figure S9). By contrast, in the presence of Pd nanozymes together with H_2_O_2_, the DNA strands showed extensive fragmentation and shortening. In these AFM images, DNA degradation was identified by the loss of long continuous filaments and network connectivity, together with the appearance of short fragmented features in both 2D height maps and 3D topographic profiles. Circular dichroism (CD) spectroscopy further revealed pronounced spectral alterations upon Pd nanozymes treatment (Figure 3B). The characteristic absorption peaks of DNA at 250 nm and 278 nm underwent noticeable intensity changes, accompanied by slight spectral shifts. The reduction in ellipticity at 278 nm, corresponding to base stacking and helical conformation, suggests that Pd nanozymes interact strongly with DNA bases and disrupt its secondary structure. This structural perturbation facilitates cleavage of the phosphodiester backbone and thereby promotes DNA degradation. Based on these results, a plausible degradation mechanism was proposed where Pd nanozymes catalyze H_2_O_2_ decomposition to generate ·OH, resulting in strand scission and ARGs inactivation (Figure 3C).

Agarose‐gel electrophoresis confirmed DNA degradation across different treatments (Figure 3D). Prominent band loss was observed in the Pd + H_2_O_2_, N4 + Pd + H_2_O_2_, and N4@Pd + H_2_O_2_ groups, while intact DNA bands remained in the control, H_2_O_2_ and N4‐only groups. Degradation intensity increased with Pd concentration (Figure S10), demonstrating concentration‐dependent catalytic behavior. Quantitative real‐time PCR (qPCR) further revealed a ∼3.2 log_10_ reduction in ARGs copy numbers in the Pd + H_2_O_2_, N4 + Pd + H_2_O_2_, and N4@Pd + H_2_O_2_ groups relative to the control (Figure 3E), demonstrating efficient ARGs inactivation. These results provide direct evidence for the catalytic degradation of plasmid‐borne *bla*_NDM‐1_, illustrating that the nanorobot disrupts the structural and genetic reservoir of antibiotic resistance.

The antibacterial activity of the N4@Pd nanorobot was assessed under different treatment conditions (Figures S11 and S12). Even at a low concentration of N4 (1 × 10^7^ PFU·mL^−1^) and Pd (50 ng·mL^−1^), strong antibacterial effects were observed, as shown by viability assays (Figure 3F) and the corresponding plate images (Figure S13). The N4@Pd nanorobot reduced bacterial viability to below the detection limit of the plate‐counting assay (LOD = 10 CFU·mL^−1^) (mean ± SD, n = 3), highlighting its strong catalytic and biological synergy. The ROS measurements further confirmed the underlying oxidative mechanism. Intracellular ROS fluorescence increased most prominently in the N4@Pd nanorobot group (Figure 3G), correlating with its superior bactericidal activity. Additional biochemical assays demonstrated the downstream impact of oxidative damage. Protein leakage assays showed substantial cytoplasmic release (Figure S14), while elevated lipid‐peroxidation levels (Figure 3H) and depleted intracellular ATP (Figure S15) indicated severe membrane damage and metabolic collapse.

Collectively, the Pd component within the N4@Pd nanorobot generates ROS that simultaneously damages bacterial cells and degrades plasmid‐borne *bla*_NDM‐1_ genes. The N4@Pd nanorobot induces membrane disruption, protein leakage, lipid peroxidation and ATP depletion, ultimately causing extensive bacterial inactivation. At the same time, Pd‐catalyzed ·OH production promotes DNA strand scission, leading to a significant reduction in ARGs copy numbers. The combined effects on both bacterial viability and genetic integrity demonstrate that the N4@Pd nanorobot is capable of eliminating multidrug‐resistant cells while degrading their resistance genes. By simultaneously eliminating host cells and removing their resistance genes, this approach has the potential to reduce the genetic pool available for resistance development, which may in turn lessen the probability of phage resistance.

### 2.4 Removal of *E. coli* NDM‐1 Biofilms

**FIGURE 4:**
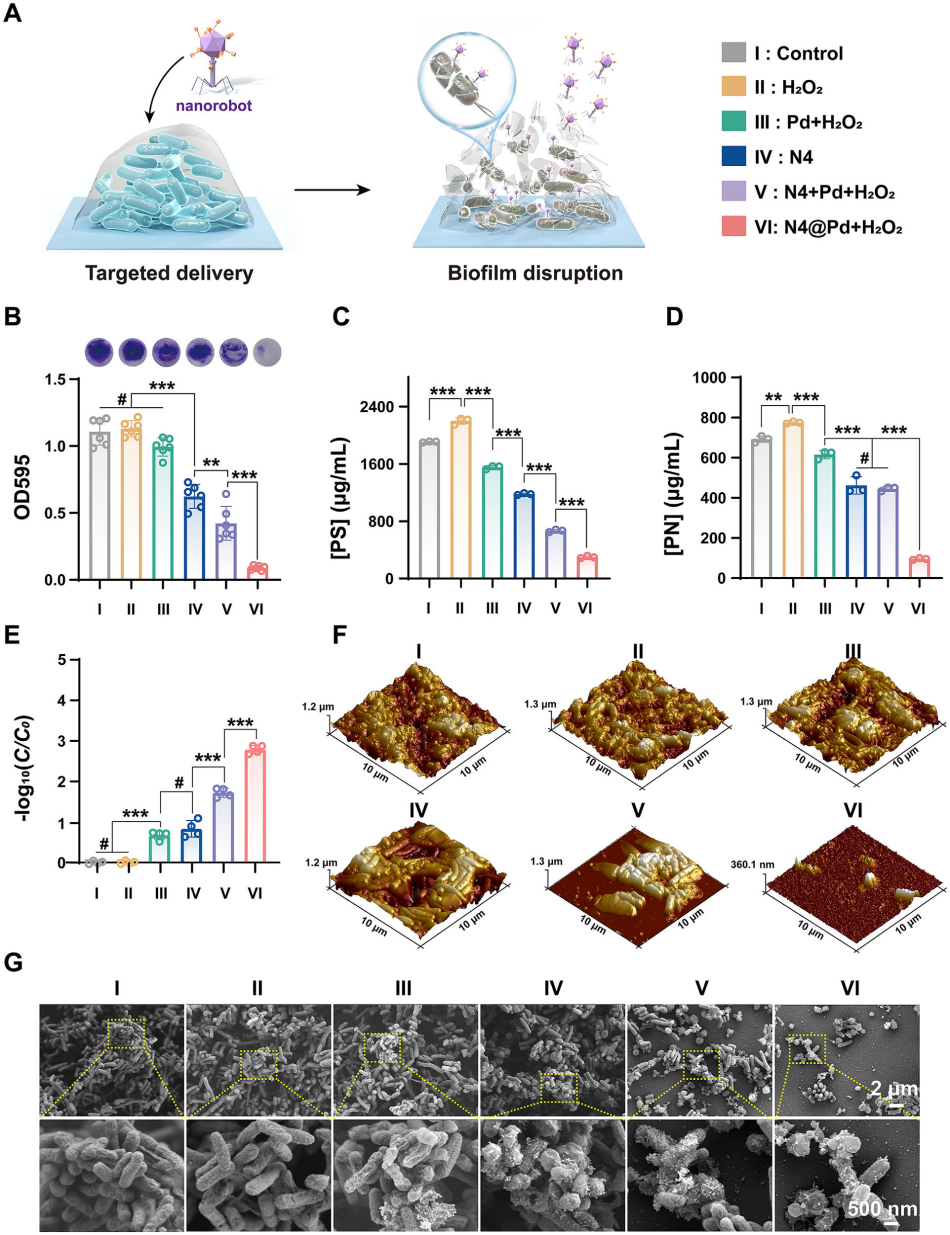
Evaluation of the antibiofilm activity of the N4@Pd nanorobot against *E. coli* NDM‐1. (A) Schematic illustration of antibiofilm mechanism of the N4@Pd nanorobot. (B) Evaluation of biofilm biomass of *E. coli* NDM‐1 under different treatments for 12 h (n = 6). (C‐D) The PS and PN contents in *E. coli* NDM‐1 biofilm after different treatments. (E) Quantitative analysis of ARGs copy number in *E. coli* NDM‐1 biofilm following each treatment. (F) 3D AFM images of *E. coli* NDM‐1 biofilm under different treatments. (G) SEM images of *E. coli* NDM‐1 biofilm under different treatments. Scale bar = 2 µm; Enlarged views Scale bar = 500 nm. Treatment groups: (I) PBS (control), (II) H_2_O_2_ (1 mM), (III) Pd (50 µg·mL^−1^) + H_2_O_2_ (1 mM), (IV) N4 (1×10^10^ PFU·mL^−1^), (V) Pd + N4 + H_2_O_2_ (same concentrations as above) and (VI) N4@Pd (containing the corresponding N4 and Pd doses) + H_2_O_2_ (1 mM). Data are presented as mean ± SD. Group differences were analyzed by one‐way ANOVA followed by Tukey's HSD post‐hoc test. Significance is denoted as *p < 0.05, **p < 0.01, ***p < 0.001, and # indicates no significant difference (*P* ≥ 0.05).

Building on the strong antibacterial and catalytic effects, the synergistic action of the phage's lytic capability and the nanozyme activity of Pd enable the N4@Pd nanorobot to eradicate mature *E. coli* NDM‐1 biofilms (Figure 4A). The antibiofilm performance was evaluated to elucidate the cooperative roles of the phage and Pd nanozyme. Crystal violet staining revealed an approximately 95% reduction in biofilm biomass after treatment, compared to the control and H_2_O_2_ groups (Figure 4B; Figure S16). To examine the compositional changes within the EPS matrix, polysaccharides (PS) and proteins (PN) levels were quantified (Figure 4C,D), which together determine biofilm stability. Pd + H_2_O_2_ or N4 alone caused partial EPS degradation, whereas the N4@Pd + H_2_O_2_ group displayed a drastic reduction in total EPS and a lower PN/PS ratio, indicating substantial disruption of the biofilm matrix by the nanorobot (Figure S17). qPCR showed the greatest reduction in *bla*_NDM‐1_ copy numbers (2.8 log_10_) in the N4@Pd + H_2_O_2_ group, higher than the Pd + H_2_O_2_ (0.7 log_10_), N4 (0.9 log_10_), or N4 + Pd + H_2_O_2_ (1.6 log_10_) (Figure 4E). These results demonstrate that the nanorobot effectively clears biofilms while simultaneously degrading released ARGs.

We characterized the morphological changes of *E. coli* NDM‐1 biofilms under different treatments (Figure 4F; Figure S18). In the control and H_2_O_2_‐only groups, continuous, uneven surfaces typical of mature biofilms were observed, whereas N4@Pd treatment produced smoother, thinner surfaces with reduced height roughness. Scanning electron microscopic (SEM) images further corroborated these findings (Figure 4G; Figure S19). The control and H_2_O_2_‐only groups exhibited dense, compact aggregates embedded in EPS, while Pd + H_2_O_2_ and N4 treatments caused only partial surface damage. In comparison, the N4@Pd + H_2_O_2_ treatment caused near‐complete collapse of the biofilm architecture, leaving scattered debris and exposed substrate. At higher magnification, minor cell‐surface irregularities were also observed in the control samples, which may be attributable to fixation, dehydration and vacuum drying in the SEM sample preparation. In contrast, cells in the N4@Pd + H_2_O_2_ treatment group appeared severely deformed and ruptured, indicating simultaneous physical lysis and oxidative attack. Confocal laser scanning microscopic (CLSM) images further provided complementary visual evidence (Figure S20). The 3D reconstructions revealed dense, thick biofilm in the control and H_2_O_2_ groups, while the N4@Pd + H_2_O_2_ treatment dramatically reduced biomass and thickness. Correspondingly, the cross‐sectional CLSM images demonstrated that the biofilm thickness in the N4@Pd + H_2_O_2_ group was substantially reduced compared to the other treatments, confirming near‐complete structural collapse (Figure S21).

These results indicate that the N4@Pd nanorobot disrupts biofilms through complementary structural and biochemical pathways. Phage‐mediated recognition promotes localized delivery of Pd nanozymes deep within the biofilm. The produced ·OH not only compromises cell membrane integrity but also induces oxidative cleavage of ARGs. This dual action, physical collapse of the biofilm matrix combined with degradation of extracellular genetic material, effectively overcomes the conventional problem in which biofilm destruction promotes secondary release and dissemination of ARGs. Furthermore, the ability to integrate lytic infection with ROS‐driven biochemical damage suggests a potential route to eliminating persister cells that are usually resilient under only antibiotic or phage treatments [49].

### 2.5 N4@Pd Nanorobot Repressed the Core Process of *E. coli* NDM‐1 Biofilm Formation

**FIGURE 5:**
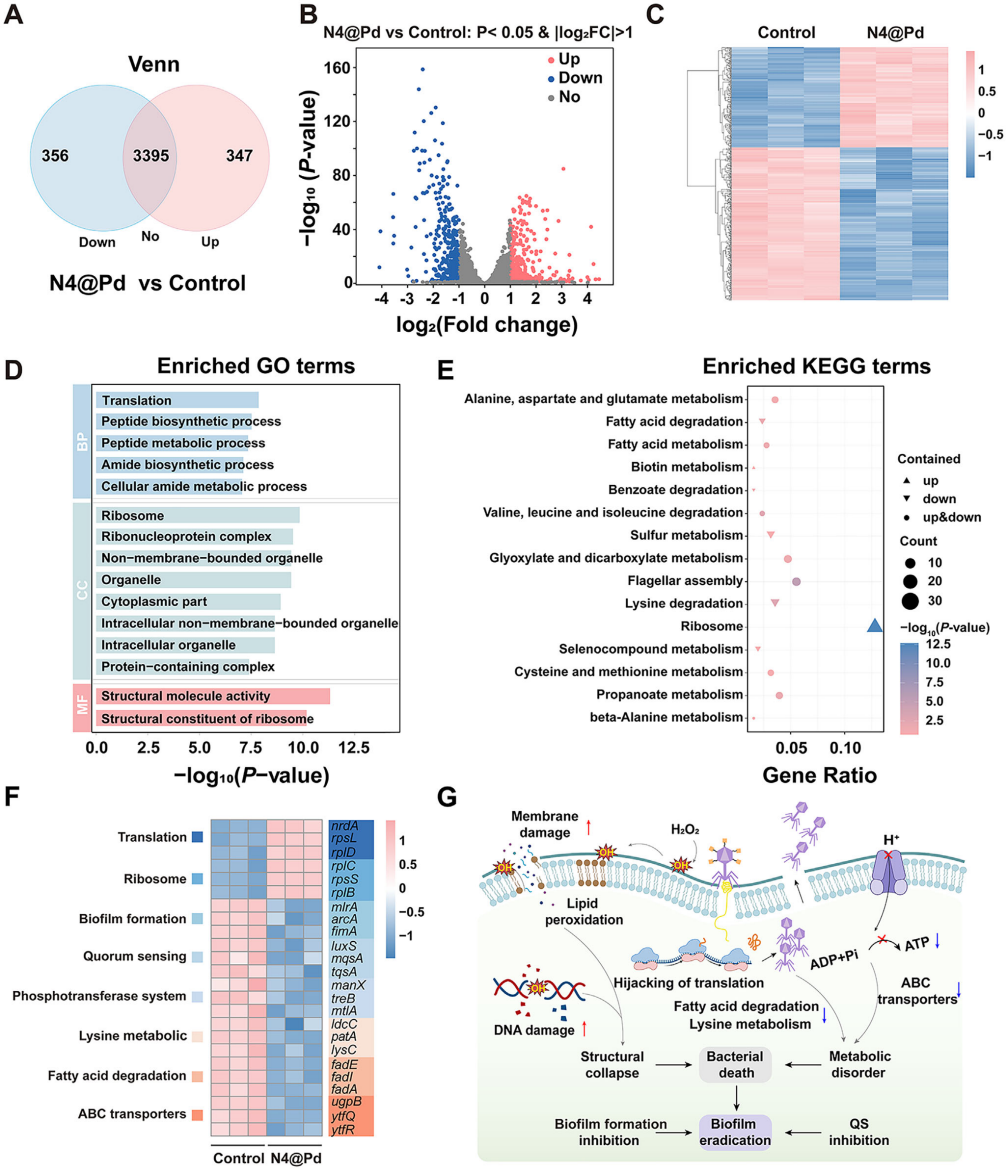
Transcriptomic analysis of *E. coli* NDM‐1 biofilm treated with PBS and N4@Pd nanorobot. (A) Venn diagram of differentially expressed genes (DEGs) in the PBS and N4@Pd nanorobot treatment groups. (B) Volcano plots of DEGs (grey: not significantly different genes; red: upregulated genes; blue: downregulated genes). The horizontal dotted line is ‐log_10_(*p*‐value) = ‐log_10_(0.05), and the vertical dashed line is |log_2_ (fold change)| = 1. (C) Cluster analysis of DEGs in control and N4@Pd nanorobot groups. Each column represents the average of three samples of each group, and each row represents a gene. Heatmap values represent relative expression levels. (D) The top 20 enriched GO terms of enriched genes under PBS and N4@Pd nanorobot treatments. (E) KEGG enrichment (top 15) of downregulated genes under PBS and N4@Pd nanorobot treatments. *p*‐value < 0.05 is considered significant. (F) Heat map of DEGs involved in bacterial metabolism pathway (blue represents genes with relatively high expression levels, red represents genes with relatively low expression levels). (G) Schematic diagram of biofilm inhibition by N4@Pd nanorobot.

To elucidate the molecular mechanisms underlying *E. coli* NDM‐1 biofilm eradication by the N4@Pd nanorobot, comparative transcriptomic analyses were performed. Differential gene expression analysis identified 356 upregulated and 347 downregulated genes in N4@Pd‐treated biofilms relative to the control (Figure 5A,B), indicating extensive transcriptomic reprogramming triggered by nanorobots exposure. Hierarchical clustering showed a separation between groups (Figure 5C), confirming the transcriptional response triggered by the nanorobot treatment. Gene Ontology (GO) enrichment analysis (Figure 5D) identified key biological processes (BP), cellular components (CC), and molecular functions (MF) affected by the treatment. Significantly enriched BP terms included translation, peptide biosynthesis, and amide metabolic processes, suggesting that the nanorobots disrupted bacterial protein synthesis machinery. Within the CC category, genes associated with ribosomes and non‐membrane‐bounded organelles were upregulated, indicating potential disturbances in ribosome function and overall cellular organization. MF enrichment further supported these findings, highlighting alterations in structural‐molecule activity, particularly related to ribosomes, being a key impacted pathway.

Kyoto Encyclopedia of Genes and Genomes (KEGG) pathway enrichment (Figure 5E) revealed multiple key target pathways modulated by the nanorobot. Downregulated pathways included fatty acid degradation, lysine degradation, phosphotransferase system (PTS), and ABC transporters, reflecting inhibited nutrient uptake, impaired energy metabolism, and disrupted membrane transport. Meanwhile, suppression of genes involved in quorum sensing (QS) and biofilm formation suggests that the nanorobot interferes with bacterial communication and adhesion processes critical for biofilm stability. In contrast, partial activation of oxidative stress‐related pathways and sulfur metabolism indicates bacterial responses to oxidative damage induced by Pd nanozyme‐mediated ROS generation. The gene‐level heatmap (Figure 5F) shows that translation‐related genes (*nrdA*, *rpsL* and *rplD*) and ribosomal assembly genes (*rplC*, *rpsS* and *rplB*) were upregulated, consistent with phage‐driven takeover of host translation machinery during infection. In contrast, key QS regulators (*luxS*, *mqsA* and *tqsA*) and biofilm formation genes (*mlrA*, *arcA* and *fimA)* were downregulated, indicating impaired communication and weakened EPS production. Moreover, genes within PTS system (*manX, treB* and *mtlA*), lysine metabolism genes (*ldcC, patA* and *lysC*), fatty acid degradation (*fadE, fadI* and *fadA*) and membrane‐associated ABC transporters (*ugpB, ytfQ* and *ytfR*) were suppressed, reflecting nutrient acquisition difficulty, metabolic stress and membrane transport inhibition under ROS pressure. These expression patterns indicate a multifaceted mechanism that simultaneously weakens bacterial biosynthesis, energy production, and intercellular signaling.

Collectively, the transcriptomic landscape reveals that the N4@Pd nanorobot interferes with QS signaling, impairs metabolic and transport processes, and hijacks host translation machinery. The multi‐level perturbation across regulatory, metabolic and structural biosynthetic pathways mechanistically accounts for the biofilm collapse and ARGs degradation (Figure 5G).

### 2.6 Performance in Complex Wastewater Environments and Ecological Impact

**FIGURE 6:**
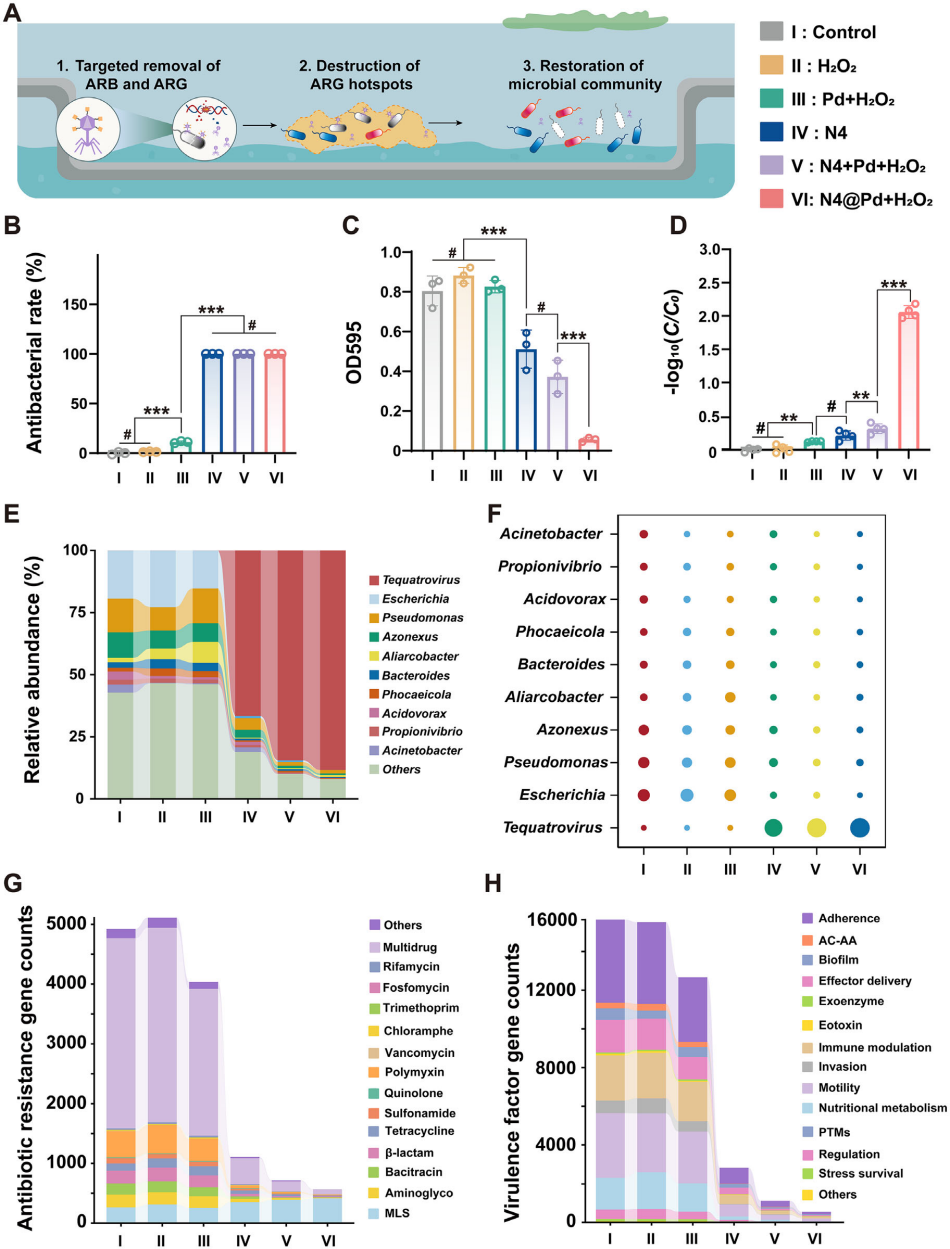
Nanorobots for synergistically eradicating antibiotic‐resistant biofilms and degradation of antibiotic resistance genes in simulated wastewater (B–D) and real sewage (E–H). (A) Schematic illustration of the N4@Pd nanorobot treatment process. (B) Antibacterial activity of different treatments in simulated wastewater for 6 h (n = 3). (C) Antibiofilm activity of different treatments in simulated wastewater for 12 h (n = 3). (D) Quantitative analysis of ARGs copy number in *E. coli* NDM‐1 biofilms treated in simulated wastewater (n = 4). (E) Relative abundance of dominant bacterial genera in each treatment group derived from metagenomic sequencing. (F) Grouped bubble chart showing the genus‐level taxonomic distribution based on metagenomic sequencing. (G) Total ARGs counts obtained from metagenomic sequencing. (H) Total virulence factor gene counts obtained from metagenomic sequencing. Treatment groups: (I) PBS (control), (II) H_2_O_2_ (1 mM), (III) Pd (50 µg·mL^−1^) + H_2_O_2_ (1 mM), (IV) N4 (1 × 10^10^ PFU·mL^−1^), (V) Pd + N4 + H_2_O_2_ (same concentrations as above), and (VI) N4@Pd (containing the corresponding N4 and Pd doses) + H_2_O_2_ (1 mM). Data are presented as mean ± SD. Group differences were analyzed by one‐way ANOVA followed by Tukey's HSD post‐hoc test. Significance is denoted as *p < 0.05, **p < 0.01, ***p < 0.001, and # indicates no significant difference (*P* ≥ 0.05).

To confirm the applicability of the N4@Pd nanorobot under complex environmental conditions, its antibacterial, antibiofilm and ARG‐degrading performance were evaluated in both simulated and actual wastewater matrices containing natural organic matter (NOM) and diverse microbial communities. The workflow of nanorobot‐mediated treatment process includes targeted removal of ARB and ARGs, destruction of ARGs hotspots (biofilms), and restoration of microbial ecosystems (Figure 6A). In simulated wastewater, more than 99% bacterial inactivation was achieved in the N4, N4 + Pd + H_2_O_2_ and N4@Pd + H_2_O_2_ groups, which were markedly superior to the control, H_2_O_2_, and Pd + H_2_O_2_ treatments (Figure 6B; Figure S22). Consistent with this, the antibiofilm experiment (Figure 6C) showed an ∼90% reduction following N4@Pd + H_2_O_2_ treatment, confirming efficient removal of bacteria and biofilms even under NOM interference. ARGs quantification further demonstrated the superior genetic deactivation performance of the nanorobots, whereas the control and H_2_O_2_ groups exhibited negligible changes. N4@Pd + H_2_O_2_ group achieved a 2.06 log_10_ reduction in *bla*_NDM‐1_ gene copy number, demonstrating a substantially enhanced capacity for both biofilm collapse and oxidative ARGs degradation in complex wastewater environments (Figure 6D). These results indicate that the nanorobot maintains strong activity under environmental complexity, enabling both biofilm collapse accompanied by oxidative ARGs degradation.

Wastewater treatment plants are reservoirs for ARGs dissemination and environmental release [50], therefore, we evaluated the environmental applicability of this nanorobot under realistic conditions. Notably, real wastewater contains diverse microbial communities that normally impose strong competition and significantly restrict ROS efficiency [51]. Therefore, we tested the nanorobots in spiked municipal wastewater, supplemented with *E. coli* NDM‐1 (∼20% of the total bacterial population) to mimic ARG‐carrying ARB under realistic environmental conditions. Metagenomic sequencing was used to profile microbial communities and ARGs dynamics after treatment (Figure 6E–H). In the control and H_2_O_2_ groups, the microbial community was dominated by *Escherichia*, *Pseudomonas*, and *Bacteroides*, confirming establishment of the target *E. coli* NDM‐1 strain (Figure 6E; Figure S23). Introduction of the phage N4 reduced *Escherichia* abundance and was accompanied by an enrichment of *Tequatrovirus* reads, indicating efficient phage infection and propagation within the complex wastewater matrix. The bubble plot (Figure 6F) and heatmap (Figure S24) illustrate this compositional shift: *Escherichia* abundance remained high in the untreated and H_2_O_2_ groups but decreased sharply in the N4, N4 + Pd + H_2_O_2_ and N4@Pd + H_2_O_2_ groups, whereas *Tequatrovirus* abundance increased, reflecting active phage replication and host suppression. In addition to targeted bacterial elimination, phage‐containing treatments significantly modulated the overall microbial structure. The N4, N4 + Pd + H_2_O_2,_ and N4@Pd + H_2_O_2_ treatments exhibited pronounced suppression of pathogenic genera (*Escherichia*, *Pseudomonas*, and *Bacteroides*) and enrichment of non‐target or potentially competitive taxa such as *Acinetobacter* and *Aliarcobacter*, suggesting partial restoration of microbial homeostasis rather than simple disinfection.

Metagenomic ARG profiling revealed a strong reduction in overall ARGs counts upon phage treatment, particularly in N4 + Pd + H_2_O_2_ and N4@Pd + H_2_O_2_ groups (Figure 6G). Notably, genes conferring multidrug, sulfonamide, and β‐lactam resistance were reduced, consistent with the targeted elimination of *E. coli* NDM‐1 and the oxidative degradation of extracellular ARGs fragments by the Pd nanozyme. Moreover, the functional gene profile analysis showed that both virulence factor genes (Figure 6H) and biocide resistance genes (Figure S25) significantly decreased in the phage‐treated groups, indicating that the combined phage‐nanozyme not only suppressed antibiotic resistance but also mitigated microbial pathogenicity and environmental resilience. Together, these results demonstrate that the N4@Pd nanorobot remains effective in complex wastewater environments, simultaneously suppressing ARB and degrading released ARGs. From an ecological perspective, the combined lytic and catalytic actions effectively restructure the microbiome by selectively removing ARGs carriers and collapsing resistance‐associated biofilm niches. Importantly, the Pd nanozyme was introduced at a concentration of 50 µg·mL^−1^ under controlled experimental conditions to evaluate catalytic performance and mechanistic effects and was immobilized via conjugation to the phage capsid, rather than being freely dispersed in the wastewater matrix. Under practical treatment conditions, such biohybrid nanostructures are expected to be retained or recoverable through conventional solid–liquid separation processes (e.g., sedimentation, filtration, or sludge capture), thereby mitigating the potential environmental impact of residual Pd at the proof‐of‐concept stage. Given the increasing concern over ARGs propagation across wastewater effluents, membrane bioreactors, and distribution pipelines, this dual‐function platform provides a practically relevant and scalable strategy for integrated biofilm control, microbiome modulation and resistance mitigation in real‐world water treatment scenarios.

## 3 Conclusion

We developed a bifunctional N4@Pd nanorobot that integrates the host specificity of bacteriophages with the catalytic activity of Pd nanozyme for precise elimination of multidrug‐resistant *E. coli* NDM‐1 biofilms. The phage N4 enables targeted bacterial recognition and lysis, while the Pd nanozyme generates ROS to enhance bactericidal efficacy and oxidatively degrade plasmid‐encoded ARGs. The nanorobot effectively disrupts bacterial metabolism and biofilm architecture, achieving simultaneous biofilm removal and ARGs inactivation. Importantly, it maintains strong antibacterial and gene‐degrading performance in complex wastewater environments rich in NOM. This work demonstrates a robust and eco‐adaptive nanorobot platform that couples biological targeting with catalytic disinfection, offering a sustainable strategy to control antibiotic‐resistant bacteria and limit the environmental dissemination of ARGs in aquatic ecosystems.

Despite these promising outcomes, certain limitations should be acknowledged. Although the Pd nanozyme was activated under a relatively low H_2_O_2_ concentration (1 mM, equivalent to 34 mg·L^−1^) in this study, sustained external oxidant supply may still pose practical challenges for large‐scale or fully autonomous wastewater treatment applications. Future designs may incorporate self‐fueled propulsion or light‐driven catalytic regeneration to achieve true self‐powered nanorobotic functionality. Furthermore, although phage N4 exhibits a broader host range, natural biofilms often contain diverse bacteria beyond the targeted *E. coli* strains, necessitating the exploration of multi‐phage cocktails or programmable phage engineering. Long‐term ecological impacts, including effects on benign microbial populations and potential resistance development, also warrant further investigation. Future research could focus on developing multi‐phage‐nanozyme assemblies, engineering nanozymes with improved catalytic efficiencies under environmental conditions, and integrating nanorobot delivery into continuous‐flow treatment systems.

## 4 Experimental Section

### 4.1 Bacterial Cultivation

*E. coli* NDM‐1 (ATCC BAA‐2452), carrying a plasmid encoding the *bla*_NDM‐1_ resistance determinant, was selected as the representative strain for assessing the antibacterial and antibiofilm performance of the N4@Pd nanorobot. Bacterial stocks were maintained on Luria‐Bertani (LB) agar plates and propagated in LB medium at 37°C under standard incubation conditions. For liquid cultivation, individual colonies were transferred into LB broth and cultured at 37°C with continuous agitation (150 rpm) overnight. When required, ampicillin was supplemented at a final concentration of 100 µg·mL^−1^ to maintain plasmid stability.

### 4.2 Isolation and Characterization of Bacteriophage N4

Bacteriophages were recovered from wastewater samples using a double‐layer agar (DLA) enrichment approach [52]. Following removal of particulate matter by centrifugation and membrane filtration (0.22 µm), the clarified samples were combined with *E. coli* NDM‐1 in 2× LB medium and incubated at 37°C with agitation for phage enrichment. Following centrifugation (10,000 rpm, 10 min), the phage‐containing supernatant was purified. Three phages were isolated, namely N1, N2, and N4. The lytic polyvalent phage N4, designated vB_EcoM_HZ_ZJUN4 was selected for subsequent experiments. Phage morphology was examined by TEM following negative staining with phosphotungstic acid. The biological properties of phage N4 were systematically evaluated, including its optimal multiplicity of infection, tolerance to temperature and pH variations, growth kinetics, and host range.

### 4.3 Synthesis of Pd Nanozymes

Pd nanozymes were synthesized via a hydrothermal method as previously described [41, 53] with slight modification. Briefly, poly(vinylpyrrolidone) (PVP, 0.42 g), ascorbic acid (AA, 0.12 g), and KBr (0.6 g) were dissolved in ultrapure water (32 mL) to form a homogeneous solution, which was heated to 80°C under stirring. An aqueous solution of K_2_PdCl_4_ (0.26 g in 12 mL) was subsequently introduced, and the reaction was maintained at 80°C in air for 3 h. After cooling to ambient temperature, the resulting Pd nanozymes were collected, washed with acetone and a mixture of 80 mg·mL^−1^ NaCl aqueous solution and acetone (3:5 v/v) to remove residual stabilizers, and finally dispersed in ultrapure water for storage at 4°C. The peroxidase‐like activity of Pd nanozymes was evaluated using UV–vis spectroscopy.

### 4.4 Construction and Optimization of N4@Pd Nanorobot

Prior to conjugation, the effects of HS‐PEG‐NHS on both phage infectivity and nanozyme activity were evaluated. A volume of 10 µL of HS‐PEG‐NHS (1 mg·mL^−1^) was added to 100 µL of Pd nanozymes (100 µg·mL^−1^), mixed thoroughly and incubated in ice bath for 30 min. Subsequently, phage N4 (1 × 10^10^ PFU·mL^−1^) was introduced and incubated for an additional 2 h to allow phage conjugation. Afterward, PEG‐NaCl (20% w/v PEG‐6,000 in 2.5 M NaCl) solution was added at a final ratio of 1:4 (v/v), and the mixture was incubated for 1 h on ice. The assembled N4@Pd nanorobots were collected by centrifugation and washed to remove unbound components. Successful formation of the N4@Pd nanorobot was verified by TEM and quantitative inductively coupled plasma mass spectrometry (ICP‐MS) analysis.

### 4.5 Evaluation of ARGs Degradation by N4@Pd Nanorobot

The ARGs degradation capability of the N4@Pd nanorobot was evaluated using plasmid DNA [pET‐29a(+)] as a model extracellular genetic substrate. Depending on the analytical method, different treatment groups were applied. For AFM imaging and CD spectroscopy, plasmid DNA was treated with PBS (Control) or Pd nanozymes (50 µg·mL^−1^) + H_2_O_2_ (1 mM). AFM was used to visualize morphological disruption and fragmentation of plasmid DNA, while CD spectroscopy was used to monitor changes in DNA secondary structure. For DNA integrity and ARGs degradation analyses, plasmid DNA samples were treated under the indicated conditions and then subjected to agarose gel electrophoresis and qPCR. Gel electrophoresis was used to evaluate DNA cleavage and structural integrity, while qPCR was performed to quantify the residual abundance of the target resistance gene. Detailed experimental procedures and group settings for each assay are provided in (Section S1.7).

### 4.6 Assessment of Antibacterial Activity

*E. coli* NDM‐1 was cultured to the exponential growth phase and harvested by centrifugation at 6,000 rpm for 5 min. The bacterial pellet was resuspended in PBS and diluted to a final concentration of 1 × 10^8^ CFU·mL^−1^. To assess the antibacterial capability, six treatment groups were tested: (I) PBS (control), (II) H_2_O_2_ (1 mM), (III) Pd (50 ng·mL^−1^) + H_2_O_2_ (1 mM), (IV) N4 (1 × 10^7^ PFU·mL^−1^), (V) Pd + N4 + H_2_O_2_ (same concentrations as above) and (VI) N4@Pd (containing the corresponding N4 and Pd doses) + H_2_O_2_ (1 mM). Each treatment was incubated with 1 mL of the bacterial suspension at 37°C for 6 h. Following treatment, samples were washed twice with PBS to remove free phages and residual reagents, and resuspended in fresh PBS. Bacterial survival was quantified by plating serial tenfold dilutions onto LB agar followed by incubation at 37°C until colony formation. ROS levels, protein leakage, lipid peroxidation and ATP depletion were further assessed to investigate antibacterial mechanisms. All experiments were performed in triplicate.

### 4.7 Evaluation of Antibiofilm Performance

Biofilms were established by incubating *E. coli* NDM‐1 (1 × 10^6^ CFU·mL^−1^) in LB at 37°C for 24 h in sterile 24‐well plates. Following incubation, the culture medium was discarded, and loosely attached cells were carefully removed by gentle rinsing with PBS. Preformed biofilms were then treated under six conditions for 12 h: (I) PBS (control), (II) H_2_O_2_ (1 mM), (III) Pd (50 µg·mL^−1^) + H_2_O_2_ (1 mM), (IV) N4 (1 × 10^10^ PFU·mL^−1^), (V) Pd + N4 + H_2_O_2_ (same concentrations as above) and (VI) N4@Pd (containing the corresponding N4 and Pd doses) + H_2_O_2_ (1 mM). Then, biofilm biomass reduction was quantified by the crystal violet assay. EPS were extracted from the collected biofilms following a heat treatment method and subsequently quantified. Biofilm morphology and structural disruption were examined by SEM, AFM, and CLSM. SEM and CLSM were used to visualize biofilm architecture and AFM to assess surface topology.

### 4.8 Transcriptomic Analysis

*E. coli* NDM‐1 was grown for 24 h to allow biofilm formation and subsequently treated with PBS (control) or N4@Pd nanorobot for 10 min. Following treatment, 1 mM H_2_O_2_ was added to each group and incubated for 12 h. Following PBS washing, the residual biofilm sample was collected for analysis. RNA sequencing and analysis were performed by Novogene (Illumina Novaseq Xplus, Beijing, China), using three biological replicates per group. The sequencing datasets have been submitted to the NCBI Sequence Read Archive (SRA) under accession number SUB15182049. Transcript abundance was calculated using HTSeq (v0.6.1), with expression levels normalized as FPKM values. Differential gene expression analysis was conducted with DESeq2 (adjusted *p* < 0.05, |log_2_ fold change| > 1). GO and KEGG pathway analyses were carried out using the GOseq R package and KOBAS software, respectively, with significance set at *p* < 0.05.

### 4.9 Validation of Nanorobot Performance in Simulated Wastewater

Artificial wastewater was prepared following Table S1 to simulate municipal influent. Humic acid (10 mg·L^−1^) was added as a representative component of NOM to mimic matrix interference [54]. The pH was adjusted to 7.0 ± 0.2 using 10 mM PBS. *E. coli* NDM‐1 cells (1 × 10^8^ CFU·mL^−1^) were spiked into the simulated wastewater to represent ARB. To assess the antibacterial capability, six treatment groups were tested: (I) PBS (control), (II) 1 mM H_2_O_2_, (III) 50 µg·mL^−1^ Pd + 1 mM H_2_O_2_, (IV) 1 × 10^10^ PFU·mL^−1^ N4, (V) a mixture of 50 µg·mL^−1^ Pd + 1 × 10^10^ PFU·mL^−1^ N4 + 1 mM H_2_O_2_, and (VI) N4@Pd nanorobot (1 × 10^10^ PFU·mL^−1^ N4 and ∼50 µg·mL^−1^ Pd) + 1 mM H_2_O_2_. Each treatment was incubated with 1 mL of the bacterial suspension at 37°C for 6 h. Following treatment, samples were serially diluted, plated on LB agar and incubated overnight to quantify surviving bacteria. For antibiofilm assays, biofilms were established by incubating *E. coli* NDM‐1 (1 × 10^6^ CFU·mL^−1^) in simulated wastewater at 37°C for 24 h in sterile 24‐well plates. The preformed biofilms were then treated with the same six experimental groups at 37°C for 12 h, and biofilm biomass was quantified using the crystal violet assay. In parallel, biofilms established and treated under the same conditions were collected for quantitative analysis of ARGs by qPCR.

### 4.10 Validation of Nanorobot Performance in Real Wastewater

To further evaluate environmental applicability, raw influent was collected from a municipal wastewater treatment plant in Hangzhou, China. The influent, representative of typical urban domestic sewage, was filtered through 0.45 µm membranes to remove suspended solids, and spiked with *E. coli* NDM‐1 (2 × 10^5^ CFU·mL^−1^). Samples were then subjected to the same conditions described in Section 4.9 for 6 h. After treatment, samples were stored at ‐80°C for subsequent metagenomic analysis. Metagenomic sequencing and 16S rRNA gene sequencing were performed by Shanghai LingEn Biotechnology Co., Ltd. (Shanghai, China).

### 4.11 Statistical Analysis

All experiments were performed with at least three independent replicates unless otherwise specified. Data are presented as mean ± standard deviation (SD), and the specific sample size (n) for each experiment is indicated in the corresponding figure legends. Figures in this study were drawn using Origin 2022, GraphPad Prism 8.0, and the online platform Chiplot (https://www.chiplot.online). Statistical analyses were performed in SPSS 26.0, using one‐way ANOVA, followed by Tukey's HSD post‐hoc test with multiplicity‐adjusted *P*‐values. Significance was indicated as *p < 0.05, **p < 0.01, ***p < 0.001, and # indicates no significant difference (*P* ≥ 0.05).

## Author Contributions

J.Z.: conceptualization, methodology, data curation, formal analysis, visualization, writing – original draft. T.D.: investigation, funding acquisition, visualization, writing – review, and editing. L.W.: conceptualization, methodology, formal analysis, funding acquisition, writing – review and editing, supervision, project administration. J.Y.: formal analysis, supervision, visualization, writing – review, and editing. W.W.: investigation, methodology. S.G.: visualization. X.L.: visualization. X.H.: visualization. Y.X.: supervision, funding acquisition, visualization, resources, writing – review, and editing. D.W.: supervision, funding acquisition, resources, writing – review, and editing. All authors have read and approved the final version of the manuscript.

## Conflicts of Interest

The authors declare no conflicts of interest.

## Supporting information




**Supporting File**: advs75287‐sup‐0001‐SuppMat.docx.

## Data Availability

The data that support the findings of this study are available from the corresponding author upon reasonable request.
