# Supplementary material for: A Dual‐Functional Biohybrid Nanorobot to Synergistically Eradicate Biofilm and Degrade Antibiotic Resistance Genes
Source: Adv Sci (Weinh). 2026 Apr 14;13(39):e75287. doi: 10.1002/advs.75287 (PMC13334968; doi:10.1002/advs.75287)
Supplement: Supplementary file 1 — Supporting File: advs75287‐sup‐0001‐SuppMat.docx. [file ADVS-13-e75287-s001.docx]

Supporting Information for

**A Dual-functional Biohybrid Nanorobot to Synergistically Eradicate Biofilm and Degrade Antibiotic Resistance Genes**

Junzheng Zhang^1,2^, Tong Dou^1,2^, Luokai Wang^1,3*^, Jingxian Yang^4^, Wenrui Wang^1^, Shuaitian Guo^1^, Xiangyu Li^1^, Xuefang Huang^5^, Yunlei Xianyu^6,7*^, Dongsheng Wang^1,2*^

^1^ College of Environmental and Resource Sciences, Zhejiang University, Hangzhou 310058, China

^2^ Innovation Center of Yangtze River Delta, Zhejiang University, Jiashan 314100, China

^3^ School of Clinical and Basic Medical Sciences, Shandong First Medical University & Shandong Academy of Medical Sciences, Jinan 250117, China

^4^ Division of Molecular Bacterial Epidemiology & Infectious Diseases, Institute of Veterinary Bacteriology, University of Bern, Bern 3012, Switzerland

^5^ Institute of Biotechnology, Zhejiang University, Hangzhou 310058, China

^6^ Department of Clinical Laboratory, Sir Run Run Shaw Hospital, Zhejiang University School of Medicine, Hangzhou 310016, China

^7^ College of Biosystems Engineering and Food Science, Zhejiang Key Laboratory of Agro-food Resources and High-value Utilization, Zhejiang University, Hangzhou 310058, China

*Corresponding authors:

Luokai Wang,

Email: lkwang@sdfmu.edu.cn

Yunlei Xianyu,

Email: xianyu19@zju.edu.cn

Dongsheng Wang,

Email: [wongds@zju.edu](mailto:wongds@zju.edu).cn

**Content**

[1. Materials and methods 1](#_Toc224048846)

[1.1 Chemicals and reagents 1](#_Toc224048847)

[1.2 Instrumentation 2](#_Toc224048848)

[1.3 Phage purification and propagation 3](#_Toc224048849)

[1.4 Physiological characterization of bacteriophage N4 4](#_Toc224048850)

[1.5 Measurement of peroxidase-like activities of Pd nanozymes 6](#_Toc224048851)

[1.6 Assessment of the impact of HS-PEG-NHS on phage viability and Pd nanozyme activity 7](#_Toc224048852)

[1.7 ICP-MS-based determination of conjugation efficiency 8](#_Toc224048853)

[1.8 Characterization of plasmid structure 10](#_Toc224048854)

[1.9 Quantification of *bla*_NDM-1_ gene in plasmid [pET-29a(+)] 11](#_Toc224048855)

[1.10 Measurement of ROS in the *E. coli* NDM-1 11](#_Toc224048856)

[1.11 Measurement of LPO in the *E. coli* NDM-1 12](#_Toc224048857)

[1.12 Measurement of protein leakage in the *E. coli* NDM-1 13](#_Toc224048858)

[1.13 Measurement of ATP in the *E. coli* NDM-1 13](#_Toc224048859)

[1.14 Evaluation of biofilm removal efficiency 14](#_Toc224048860)

[1.15 Extracellular polymeric substances (EPS) extraction 14](#_Toc224048861)

[1.16 Preparation of biofilm samples for SEM 15](#_Toc224048862)

[1.17 Preparation of biofilm samples for AFM 15](#_Toc224048863)

[1.18 Preparation of biofilm samples for CLSM imaging 16](#_Toc224048864)

[2. Supporting figures and tables 17](#_Toc224048865)

[3. References 43](#_Toc224048866)

# 1. Materials and methods

## 1.1 Chemicals and reagents

All chemical and biological reagents were purchased from commercial suppliers without further purification. Potassium palladium (II) chloride (K_2_PdCl_4_, ≥ 99.99%), sodium chloride (NaCl, ≥ 99.5%), polyethylene glycol (PEG, Mw = 6 kDa), poly (vinyl pyrrolidone) (PVP, Mw = 55 kDa), L-ascorbic acid (AA, >98.0%), 3,3’,5,5’-tetramethylbenzidine (TMB, ≥ 99%), crystal violet nonahydrate and potassium bromide (KBr) were purchased from Shanghai Aladdin Biochemical Technology Co., Ltd. Sulfuric acid (H_2_SO_4_, 95.0-98.0%), methanol (≥ 99.5%), absolute ethanol (≥ 99.7%), phenol (≥ 99.0%), acetic acid (≥ 99.5%) and acetone (≥ 99.5%) were obtained from Sinopharm Chemical Reagent Co., Ltd. Heterobifunctional polyethylene glycol bearing terminal thiol and N-hydroxysuccinimide groups (HS-PEG-NHS, Mw = 1 kDa, mono-functional NHS ester, degree of substitution ≈ 1) was purchased from Shanghai Ponsure Biological Technology Co., Ltd. Plasmid extraction kit (DP104) was purchased from Tiangen Biotech Co., Ltd. Luria-Bertani (LB) broth and LB agar, as well as phosphate-buffered saline (PBS), were obtained from Solarbio Life Sciences Co., Ltd. Intracellular ROS levels were determined using a Reactive Oxygen Species Assay Kit (Solarbio, China) based on the fluorescent probe 2',7'-dichlorodihydrofluorescein diacetate (DCFH-DA). The lipid peroxide (LPO) was detected by LPO Content Assay Kit (Cat#BC5245, Solarbio, China). The Bicinchoninic Acid Protein Assay Kit (Cat#PC0020; Solarbio, China) was used to detect protein leakage from bacteria induced by different treatments [1]. Bacterial viability was monitored using the LIVE/DEAD BacLight Bacterial Viability Kits (Cat#L13152, Thermo Fisher, USA). The deionized water used in this study was purchased from Wahaha Co., Ltd.

## 1.2 Instrumentation

Ultraviolet-visible (UV-vis) absorption spectra were recorded using a UV-vis spectrophotometer (UV-2600i, Shimadzu, Japan). The morphology and structural characteristics of the Pd nanozymes were examined by transmission electron microscopy (TEM, Talos F200X G2, FEI, USA), while the bacteriophage N4 was visualized using TEM (JEM-F200, JEOL, Japan). The crystalline phase of the Pd nanocrystals was analyzed by X-ray diffraction (XRD, D8 Advance, Bruker, Germany). Bacterial biofilms were analyzed by field-emission scanning electron microscopy (SEM, Gemini SEM 360, ZEISS, Germany; VEGA3, TESCAN, Czech Republic) and **three-dimensional biofilm architecture was further visualized using Confocal laser scanning microscopy (CLSM, LSM 880, ZEISS, Germany).** The elemental composition was determined using inductively coupled plasma mass spectrometry (ICP-MS, Agilent 8900, Agilent, USA) and surface valence states were analyzed by X-ray photoelectron spectroscopy (XPS, K-Alpha, Thermo Scientific, USA). The generation of ROS was confirmed by electron spin resonance (ESR, EMXplus-6/1, Bruker, Germany). The nanoscale surface topology of biofilms was further characterized by atomic force microscopy (AFM, Dimension Icon, Bruker, Germany). Circular dichroism (CD) spectroscopy (Chirascan, Brighttime, UK) was used to evaluate conformational changes in plasmid DNA during degradation. The zeta potential and hydrodynamic size of nanoparticles were measured using a nanoparticle size analyzer (Nano-lab, Malvern, UK). Plasmid concentration was quantified using a micro-volume spectrophotometer (Nano-300, Allsheng, China), and gel images were recorded by a gel imaging system (Tanon 1600, Tanon, China). Quantitative real-time PCR (qPCR) analyses were performed on a CFX Connect Real-Time PCR System (Bio-Rad, USA). The transcriptomic sequencing was conducted on the Illumina NovaSeq X Plus platform (Illumina, USA) with paired-end 150 bp (PE150) reads.

## 1.3 Phage purification and propagation

Briefly, 100 μL of phage stock and an overnight culture of Escherichia coli (E. coli) were mixed and spread onto the surface of a double-layer agar (DLA) plate, which was then incubated until phage-induced plaques formed. Following this, all plaques were collected and propagated in separate cultures with *E. coli* for 4 h. Both the phages and *E. coli* NDM-1 underwent the DLA assay, with the plates incubated until distinct plaques became visible on the bacterial lawn. Well-defined plaques were carefully picked. The plaques were co-incubated with *E. coli*, and plaque picking was repeated three times to obtain a purified phage population.

N4 was amplified using *E. coli* NDM-1 as the host. A single colony of *E. coli* was inoculated into LB and cultured overnight at 37 °C with shaking at 180 rpm, followed by dilution (1:100) into fresh medium and incubation to mid-logarithmic phase. The host culture was then infected with phage N4 at a multiplicity of infection of 0.01 and incubated at 37 °C with shaking until complete lysis occurred, typically within 6 h. Chloroform (0.1 mL per 50 mL culture) was added to terminate bacterial growth, and the lysate was clarified by centrifugation (10,000 rpm, 10 min, 4 °C) followed by filtration through a sterile 0.22 µm membrane. The clarified lysate was subsequently mixed with PEG-NaCl (20% w/v PEG-6000 in 2.5 M NaCl) solution and incubated in an ice-water bath overnight to precipitate the phages. The mixture was centrifuged at 10,000 g for 10 min at 4 °C, and the pellet was resuspended in SM buffer. The suspension was further centrifuged at 12000 rpm for 5 min, and the supernatant was collected as a high-concentration phage N4 stock. Phage titers were determined using the DLA method.

## 1.4 Physiological characterization of bacteriophage N4

1.4.1 Multiplicity of infection (MOI)

The optimal MOI of phage N4 was determined by infecting *E. coli* NDM-1 cultures (1×10^8^ CFU·mL^-1^) with different phage concentrations ranging from 1×10^4^ to 1×10^10^ PFU·mL^-1^, corresponding to MOI values of 0.0001-100. After incubation at 37 °C for 8 h with shaking (150 rpm), cultures were centrifuged (8,000 rpm, 10 min), and the phage titer in the supernatant was determined using the DLA method. The MOI that resulted in the highest phage yield was defined as the optimal MOI.

1.4.2 Thermal stability

Phage suspensions (10^9^ PFU·mL^-1^) were incubated at 40, 50, 60, 70, and 80 °C for 60 min. Residual titers were determined by serial dilution and plating using the DLA method.

1.4.3 pH stability

Phage suspensions were exposed to 10 mM sodium acetate or LB medium adjusted to pH 2-13 at 37 °C for 1 h. Surviving phages were quantified by the DLA method after serial dilution.

1.4.4 One-step growth curve

Exponentially growing *E. coli* NDM-1 (10^8^ CFU·mL^-1^) was infected with N4 at an MOI of 0.1 and incubated at 37 °C with shaking. Samples were taken at predetermined intervals (0-480 min), treated with chloroform, centrifuged, and filtered. Phage titers were measured to determine the latent period and burst size.

**1.4.5 Phage host-range determination**

The host range of bacteriophage N4 was determined using the spot assay method against multiple *E. coli* strains available in our laboratory, including both MDR and non-MDR strains. Briefly, bacterial strains were cultured to the logarithmic growth phase and then mixed with molten soft agar (0.3%, w/v) before being poured onto LB agar plates to form a uniform bacterial lawn. Ten microliters of serially diluted phage suspensions were spotted onto the surface of the bacterial lawn and incubated at 37 °C for 12-16 h. The lytic activity was evaluated based on the morphology and transparency of the resulting plaques.

The lysis ability was qualitatively graded as follows:

“+4”, completely transparent lysis zone, indicating full bacterial clearance;

“+3”, relatively transparent lysis zone with a few surviving colonies;

“+2”, semitransparent lysis zone;

“+1”, only a few isolated plaques observed;

“0”, no visible lysis except at the pipette contact point.

Each assay was performed in triplicate to ensure reproducibility.

## 1.5 Measurement of peroxidase-like activities of Pd nanozymes

The peroxidase-like activities of Pd nanozymes were measured by catalyzing the standard TMB chromogenic substrate with H_2_O_2_ following the reported method[2]. 20 μL Pd nanozyme (1 mg·mL^-1^), 20 μL TMB (10 mg·mL^-1^) and 20 μL H_2_O_2_ (1 M) were added sequentially to NaAc/HAc buffer (0.2 M pH = 5.5). The absorbance at 652 nm was measured after 120 s of reaction.

Similarly, the kinetic parameters (*K_m_* and *V_max_*) were evaluated using a colorimetric assay based on the oxidation of TMB in the presence of H_2_O_2_. Briefly, a reaction mixture containing 160 μL of NaAc/HAc buffer (0.2 M, pH = 5.5), 20 μL of TMB solution (10 mg·mL^-1^), 10 μL of H_2_O_2_ (0-100 mM) and 10 μL of the Pd nanozyme (1 mg·mL^-1^) was incubated at 25 °C. The absorbance at 652 nm was recorded every 30 s for 5 min using a microplate reader. The initial reaction rates were determined from the linear region of absorbance change over time.

The Michaelis-Menten kinetic parameters, including the Michaelis constant (*K_m_*) and maximum reaction velocity (*V_max_*), were obtained by fitting the initial velocity (*V_0_*) versus substrate concentration ([S]) using the Michaelis-Menten equation:

$$\text{V}\text{0}\text{ =}\frac{\text{V}\text{max}\left[ S \right]}{\text{K}\text{m}+\left[ S \right]}$$

Additionally, a Lineweaver-Burk double-reciprocal plot was constructed to determine *K_m_* and *V_max_* using the equation:

$$\frac{\text{1}}{\text{V}\text{0}}\text{ =}\frac{\text{K}\text{m}}{\text{V}\text{max}\left[ S \right]}+\frac{\text{1}}{\text{V}\text{max}}$$

All experiments were performed in triplicate.

## 1.6 Assessment of the impact of HS-PEG-NHS on phage viability and Pd nanozyme activity

HS-PEG-NHS (1 mg·mL^-1^) was used to evaluate its effect on both phage viability and Pd nanozyme catalytic activity. To assess phage viability, HS-PEG-NHS solutions with different volumes (0, 10, 25, 50, 75, 100 μL) were added to phage N4 suspensions (1×10^10^ PFU·mL^-1^) at a 1:1 volume ratio and incubated for 2 h. The remaining infective phages were quantified using the plaque assay.

Similarly, HS-PEG-NHS (1 mg·mL^-1^) with varying volumes (0, 10, 25, 50, 75, 100 μL) was added to Pd nanozyme solutions (1 mg·mL^-1^), and the peroxidase-like activity was subsequently measured using the TMB-H_2_O_2_ colorimetric assay.

To further evaluate potential toxicity of Pd nanozymes toward phages, Pd nanozymes at different concentrations (0, 100, 250, 500, 750, 1,000 μg·mL^-1^) were mixed with phage N4 (1×10^10^ PFU·mL^-1^) at a 1:1 volume ratio and incubated for 2 h, after which phage infectivity was determined.

To assess the **specific adsorption capability** of the N4@Pd nanorobot toward its host bacterium, exponentially growing *E. coli* NDM-1 (1×10^8^ CFU·mL^-1^) were incubated under three conditions (i) PBS (control), (ii) a physical mixture of Pd nanozymes (100 μg·mL^-1^) and phage N4 (2×10^10^ PFU·mL^-1^), and (iii) N4@Pd nanorobot containing equivalent amounts of Pd and phage N4. After incubation at room temperature (25 ^o^C) for 30 min, cells were collected by centrifugation (10,000 rpm for 5 min) and subjected to Pd quantification using ICP-MS. The same procedure was applied to P. aeruginosa as non-host control. All measurements were performed in triplicate.

## 1.7 ICP-MS-based determination of conjugation efficiency

The conjugation efficiency was determined by measuring the residual Pd content in the supernatant after conjugation using ICP-MS and calculated based on the depletion of Pd from the supernatant according to the following equation:

$$Conjugation efficiency\left( \% \right)=(1-\frac{{Pd}_{supernatant}}{{Pd}_{total}})\times100\%$$

All measurements were performed in triplicate and reported as mean ± SD.

The average number of Pd nanozymes per phage particle was further estimated by normalizing the Pd amount associated with the phage fraction to the phage particle concentration. Briefly, the amount of phage-bound Pd was calculated by mass balance:

$${Pd}_{associated}={Pd}_{total}-{Pd}_{supernatant}$$

The mass of Pd associated with each phage particle was then calculated as:

$$m_{Pd/phage}=\frac{{Pd}_{associated}}{N_{phage}}$$

Where *N_phage_* is the phage particle concentration (1×10^10^ PFU·mL^-1^) used for conjugation. Assuming that the Pd nanozymes were cubic particles, the mass of a single Pd nanozyme was estimated from its average diameter measured by DLS (*d* = 29.8 ± 6.2 nm) and the density of metallic Pd (*ρ_Pd_ =* 12.02 g·cm^-3^):

$$m_{\mathrm{single}Pd}=\rho_{Pd}\times d^{3}$$

The average number of Pd nanozymes loaded per phage capsid was then obtained by:

$$N_{Pd/phage}=\frac{m_{Pd/phage}}{m_{\mathrm{single}Pd}}$$

Using this approach, the average loading was estimated to be approximately 16 Pd nanozymes per phage particle.

## 1.8 Characterization of plasmid structure

1.8.1 AFM

The morphological changes in [pET-29a(+)] plasmid samples (50 ng·μL^-1^) after treatment with Pd (50 μg·mL^-1^) + H_2_O_2_ (1 mM) were examined using AFM in tapping mode [3]. For sample preparation, an appropriate volume of treated plasmid solution was deposited onto a freshly cleaved mica substrate, allowed to adsorb, gently rinsed, and dried. AFM images were acquired under optimized scanning conditions, and structural alterations were analyzed.

1.8.2 CD spectroscopy

CD spectroscopy was used to assess the conformational changes in [pET-29a(+)] plasmid samples (50 ng·μL^-1^) following Pd (50 μg·mL^-1^) + H_2_O_2_ (1 mM) treatment [4]. The plasmid samples were dissolved in an appropriate buffer (10 mM Tris-HCl, pH 7.5). CD spectra were recorded in a 1 mm quartz cuvette over a wavelength range of 200-320 nm, with a step size of 1 nm and a scanning speed of 100 nm/min.

1.8.3 Agarose gel electrophoresis

To further probe DNA strand integrity, plasmid samples (50 ng·μL^-1^) were subjected to six experimental treatments: (I) PBS (control), (II) H_2_O_2_ (1 mM), (III) Pd (50 μg·mL^-1^) + H_2_O_2_ (1 mM), (IV) N4 (1×10^10^ PFU·mL^-1^), (V) a physical mixture of Pd and N4 + H_2_O_2_ (same concentrations as above) and (VI) N4@Pd (containing the corresponding N4 and Pd doses) + H_2_O_2_ (1 mM) at 37 °C for 6 h. DNA integrity was assessed by agarose gel electrophoresis using a 1% agarose gel prepared in 1× TAE buffer, operated at 100 V for 60 min, followed by visualization with a gel documentation system.

## 1.9 Quantification of *bla*_NDM-1_ gene in plasmid [pET-29a(+)]

The *bla*_NDM-1_ gene in plasmid [pET-29a(+)] was quantified using a real-time PCR Detection System. The primer sets were (5’-CGC CAT CCC TGA CGA TCA AA-3’) as the forward primer and (5’-CTG AGC ACC GCA TTA GCC G-3’) as the reverse primer [5]. The qPCR reaction was conducted in a 20 μL reaction system with 10 μL of SYBR Green PCR Master Mix, 0.4 μL of forward primer (10 μmol·L^-1^), 0.4 μL of reverse primer (10 μmol·L^-1^), 8.2 μL H_2_O, and 1 μL DNA. The temperature program of the qPCR reaction was as follows: denaturation at 95 °C for 10 min, followed by 45 cycles of denaturation at 95 °C for 15 s, annealing at 57 °C for 1 min, followed by a melting curve for specificity verification. Each gene was quantified in triplicate along with standard curves and negative controls with nuclease-free water. Inactivation efficiencies of *bla*_NDM-1_ gene in plasmid [pET-29a(+)] were calculated as log_10_(*C_0_/C*), where the *C*_0_ and *C* represent the initial and residual *bla*_NDM-1_ gene abundance, respectively.

## 1.10 Measurement of ROS in the *E. coli* NDM-1

Intracellular ROS levels were determined using a Reactive Oxygen Species Assay Kit based on the fluorescent probe 2',7'-dichlorodihydrofluorescein diacetate (DCFH-DA). Briefly, cells were harvested, resuspended in serum-free culture medium, and adjusted to a final concentration of 1×10^6^ CFU·mL^-1^. DCFH-DA was diluted at a ratio of 1:1000 to achieve a final concentration of 1 μmol·L^-1^ and incubated with the cells at 37 °C for 40 min. During incubation, the suspension was gently inverted every 5 min to ensure uniform probe diffusion and cellular uptake. After incubation, cells were washed three times with PBS to remove any excess extracellular DCFH-DA. Following different treatments for 30 min, fluorescence intensity was measured using a microplate reader with an excitation wavelength of 488 nm and an emission wavelength of 525 nm. All measurements were performed in triplicate.

## 1.11 Measurement of LPO in the *E. coli* NDM-1

Lipid peroxide (LPO) levels were measured using an LPO Content Assay Kit (Cat#BC5245, Solarbio). In acidic conditions, LPO degradation generates malondialdehyde (MDA), which reacts with TBA to form a stable chromogenic complex (3,5,5-trimethyl-2,4-dioxoimidazolidine) with a maximum absorbance at 532 nm. For sample preparation, E. coli NDM-1 cells were collected by centrifugation, and the supernatant was discarded. The bacterial pellet was resuspended in an extraction buffer (bacterial count: 5×10^6^ CFU·mL^-1^). The suspension was subjected to ultrasonic disruption (200 W, 3 s pulse with a 7 s interval, total duration 3 min) on an ice bath. The lysate was centrifuged at 8000 rpm for 10 min at 4 °C, and the supernatant was collected for LPO analysis. The absorbance of the reaction product was measured at 532 nm using a microplate reader.

## 1.12 Measurement of protein leakage in the *E. coli* NDM-1

The Bicinchoninic Acid Protein Assay Kit (Cat#PC0020; Solarbio) was used to detect protein leakage from bacteria induced by different treatments [1]. In brief, *E. coli* NDM-1 were washed twice with PBS (pH = 7.4) by the refrigerated centrifuge (6,000 rpm) for 5 min and diluted back in PBS to the desired working concentration at an OD600 of 0.1. A total of 150 μL of the diluted bacterial suspension was added into each well of a 96-well plate and subjected to treatment for 6 h. Then, 100 μL of the treated bacterial suspension was collected and diluted to 500 μL. The diluted bacterial solutions were then centrifuged (6,000 rpm) for 5 min by the refrigerated centrifuge. Finally, the supernatant was immediately collected and the relative protein leakage of each sample was determined by a microplate reader at OD562.

## 1.13 Measurement of ATP in the *E. coli* NDM-1

*E. coli* NDM-1 cells were collected by centrifugation at 10,000 rpm for 10 min at 4 °C, and the supernatant was discarded. The bacterial pellet was resuspended in lysis buffer at a ratio of 1×10^4^ bacterial cells per 1 μL buffer. Cell lysis was performed using ultrasonication (200 W, total duration 1 min) in an ice bath. The lysate was centrifuged at 10,000 rpm for 10 min at 4 °C, and the supernatant was transferred to a fresh microcentrifuge tube. Subsequently, 500 μL of chloroform was added, and the mixture was vortexed thoroughly. After phase separation by centrifugation at 10,000 rpm for 3 min at 4 °C, the upper aqueous phase was collected and kept on ice until analysis. The ATP concentration was determined by measuring absorbance at 340 nm using UV spectrophotometer.

## 1.14 Evaluation of biofilm removal efficiency

After incubation, the bacterial suspension was carefully removed using a micropipette, ensuring minimal disruption of the biofilm. The biofilm was then gently washed 2-3 times with PBS. After removing the PBS, the plates were inverted and air-dried. The biofilm was fixed with methanol for 30 min, followed by another 30 min of drying. Crystal violet staining (1%) was then performed for 15 min. After staining, the excess dye was removed, and the biofilm was washed three times with PBS. The plates were air-dried for 6 h in an inverted position. To quantify biofilm disruption, the retained crystal violet was dissolved in 33% acetic acid for 15 min, and the OD595 was measured using a microplate reader. The biofilm disruption rate was calculated as follows:

$$Disruption Rate=\frac{{OD}_{control}-{OD}_{treatment}}{{OD}_{control}}\times100\%$$

All experiments were performed with six replicates.

## 1.15 Extracellular polymeric substances (EPS) extraction

Briefly, the biofilm samples were incubated in a water bath at 60 °C for 40 min and subsequently cooled to ambient temperature (25 °C). The suspension was then transferred to 1.5 mL centrifuge tubes and centrifuged at 8,000 rpm for 20 min. The supernatant was collected and filtered through a 0.45 μm acetate fiber membrane to obtain the extracted EPS fraction. The total polysaccharide concentration in the EPS was determined using the phenol-sulfuric acid method, with glucose as the standard. The total protein concentration was quantified using the BCA method, with bovine serum albumin as the standard. All experiments were performed in triplicate.

## 1.16 Preparation of biofilm samples for SEM

Biofilms were grown on glass coverslips and subjected to six different treatments. Following treatment, biofilm samples were immersed in 2.5% glutaraldehyde at 4 °C for 8 h. The samples were then rinsed with PBS to remove excess glutaraldehyde. A graded ethanol dehydration series was performed using 30%, 50%, 70%, and 90% ethanol for 15 min each, followed by three sequential dehydrations in 100% ethanol for 15 min each. The dehydrated samples were then subjected to critical point drying and sputter-coated with gold.

## 1.17 Preparation of biofilm samples for AFM

The preparation of biofilm samples for AFM followed a similar protocol as for SEM. Biofilms were grown on glass coverslips, treated under six different conditions, and fixed in 2.5% glutaraldehyde at 4 °C for 8 h. After fixation, the samples were rinsed with PBS and subjected to a graded ethanol dehydration series, including 30%, 50%, 70%, and 90% ethanol for 15 min each, followed by three sequential dehydrations in 100% ethanol for 15 min each. Unlike SEM sample preparation, critical point drying was not required prior to AFM imaging.

## 1.18 Preparation of biofilm samples for CLSM imaging

*E. coli* NDM-1 biofilms were established in confocal culture dishes and subjected to different treatments. Following treatment, the biofilms were stained with a SYTO9 and propidium iodide (PI) mixture in the dark for 30 min. Fluorescence images were acquired using CLSM. The fluorescence intensity was quantified using ImageJ software to evaluate antibiofilm efficacy.

# 2. Supporting figures and tables


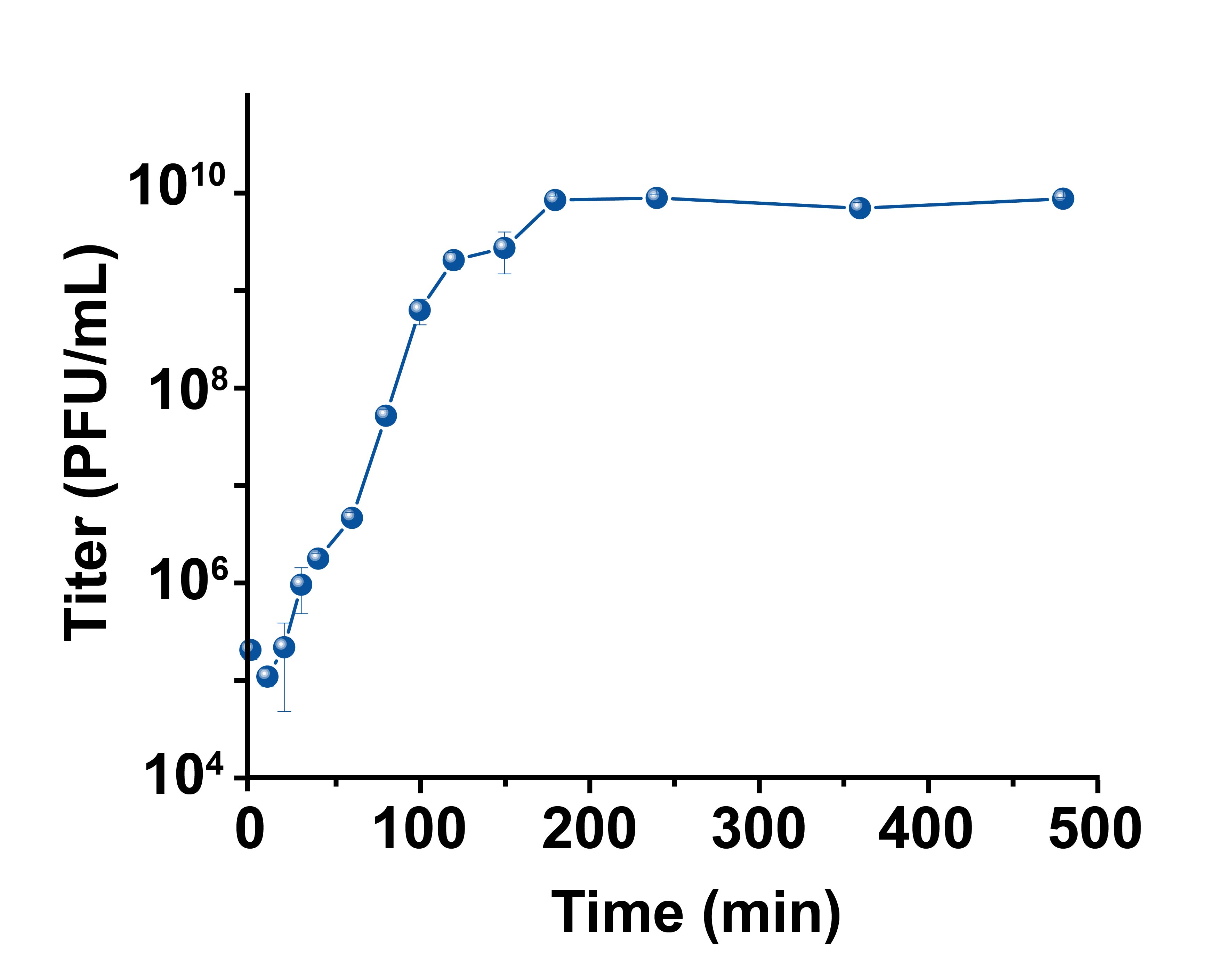


**Fig. S1** One-step growth curve of phage N4. Exponentially growing *E. coli* NDM-1 (≈1×10^8^ CFU·mL^-1^) was infected with N4 at MOI = 0.1 and incubated at 37 °C. Data are shown as mean ± SD (n = 3). All assays were performed in triplicate.


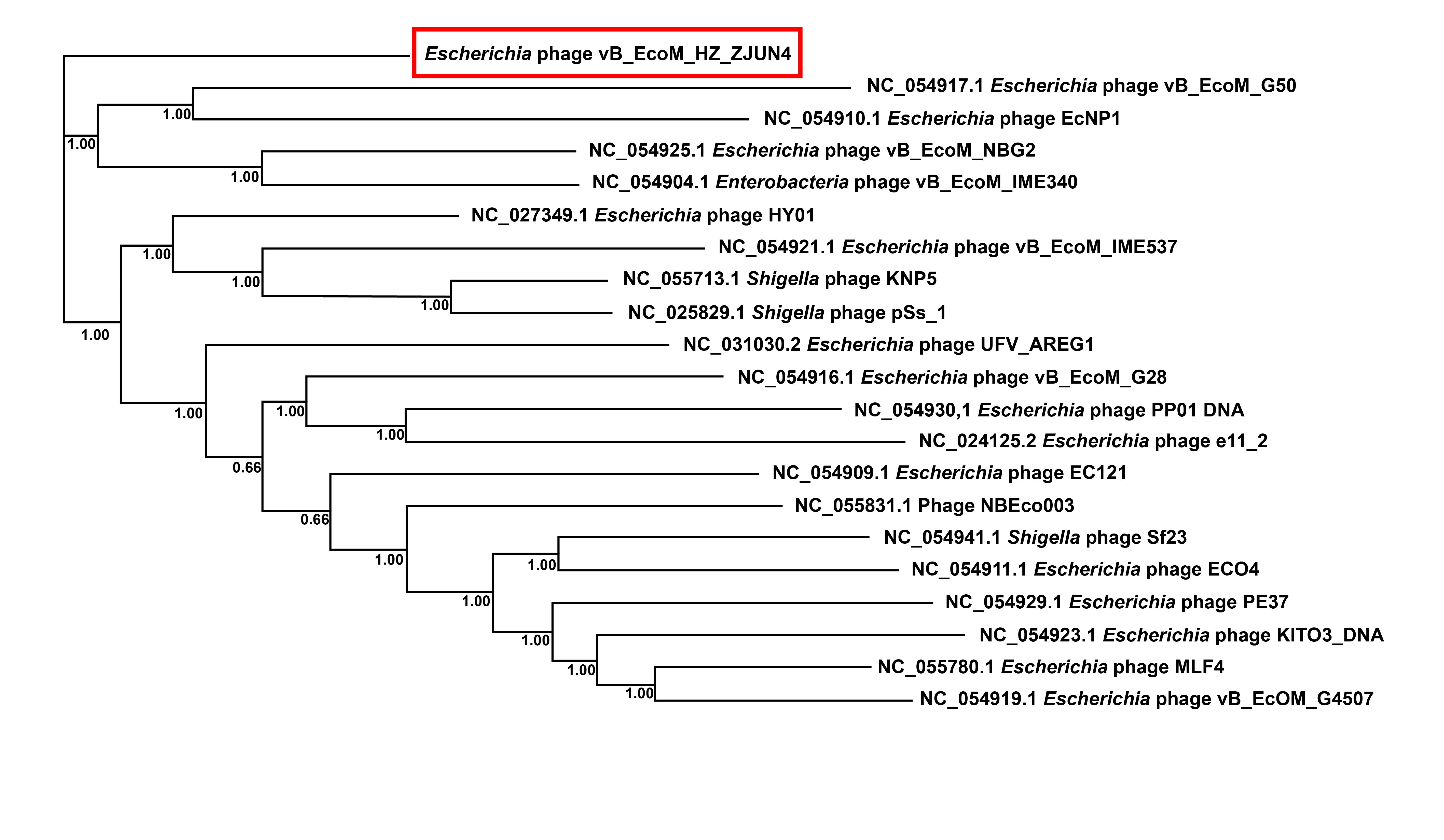
**Fig. S2** Phylogenetic analysis of phage N4 based on whole-genome sequences.


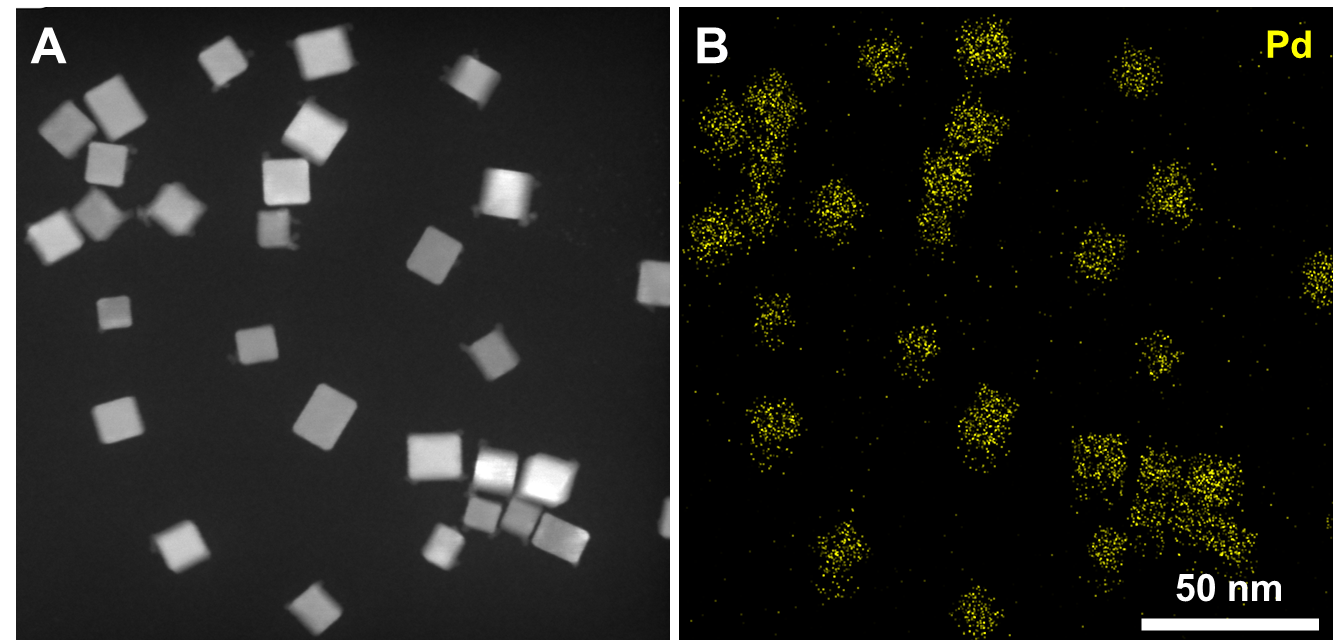


**Fig. S3** (A) HAADF-TEM image and (B) elemental EDS mapping image of Pd nanozymes. Scale bar = 50 nm.


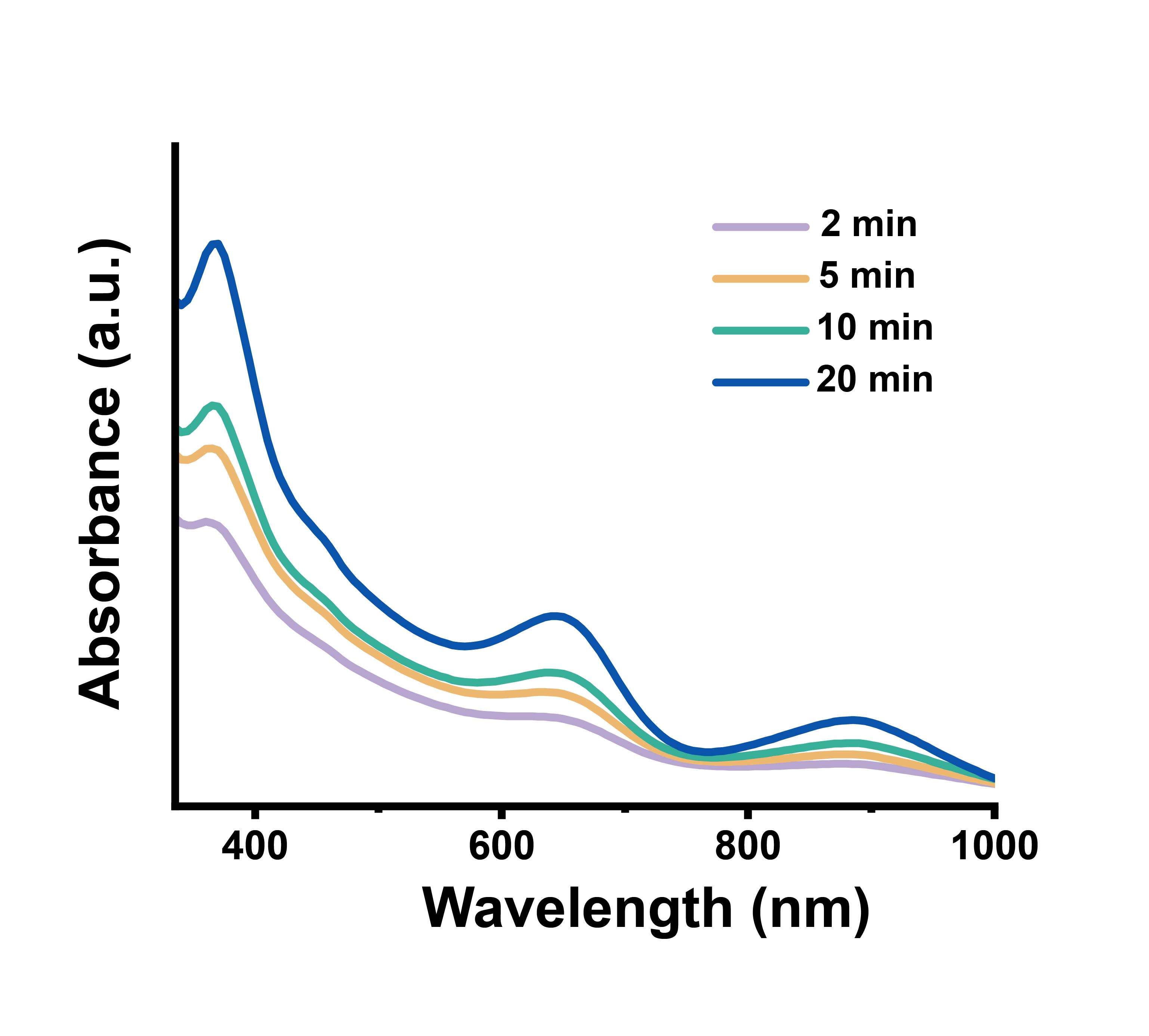


**Fig. S4** UV-Vis absorption spectra of Pd nanozymes at different reaction times (2, 5, 10 and 20 min). Experimental conditions: TMB (1 mg·mL^-1^), H_2_O_2_ (100 mM), Pd (100 μg·mL^-1^) and NaAc/HAc buffer (0.2 M, pH 5.5).


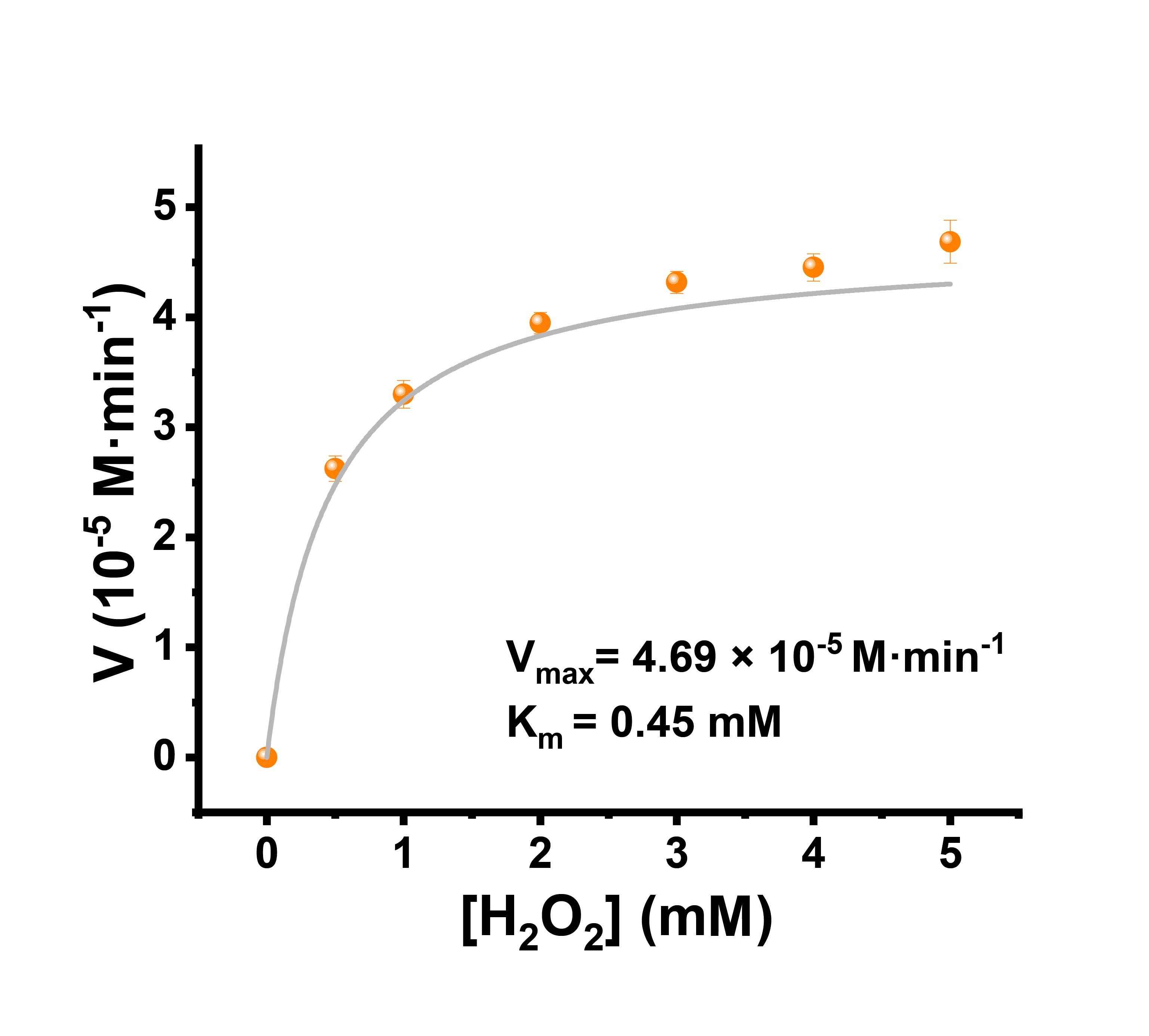


**Fig. S5** Michaelis-Menten curve for Pd nanozymes with H_2_O_2_ as a substrate in the presence of TMB at 25 ^o^C (n = 3). Experimental conditions: TMB (10 mg·mL^-1^), Pd (100 μg·mL^-1^) and NaAc/HAc buffer (0.2 M, pH 5.5).

*
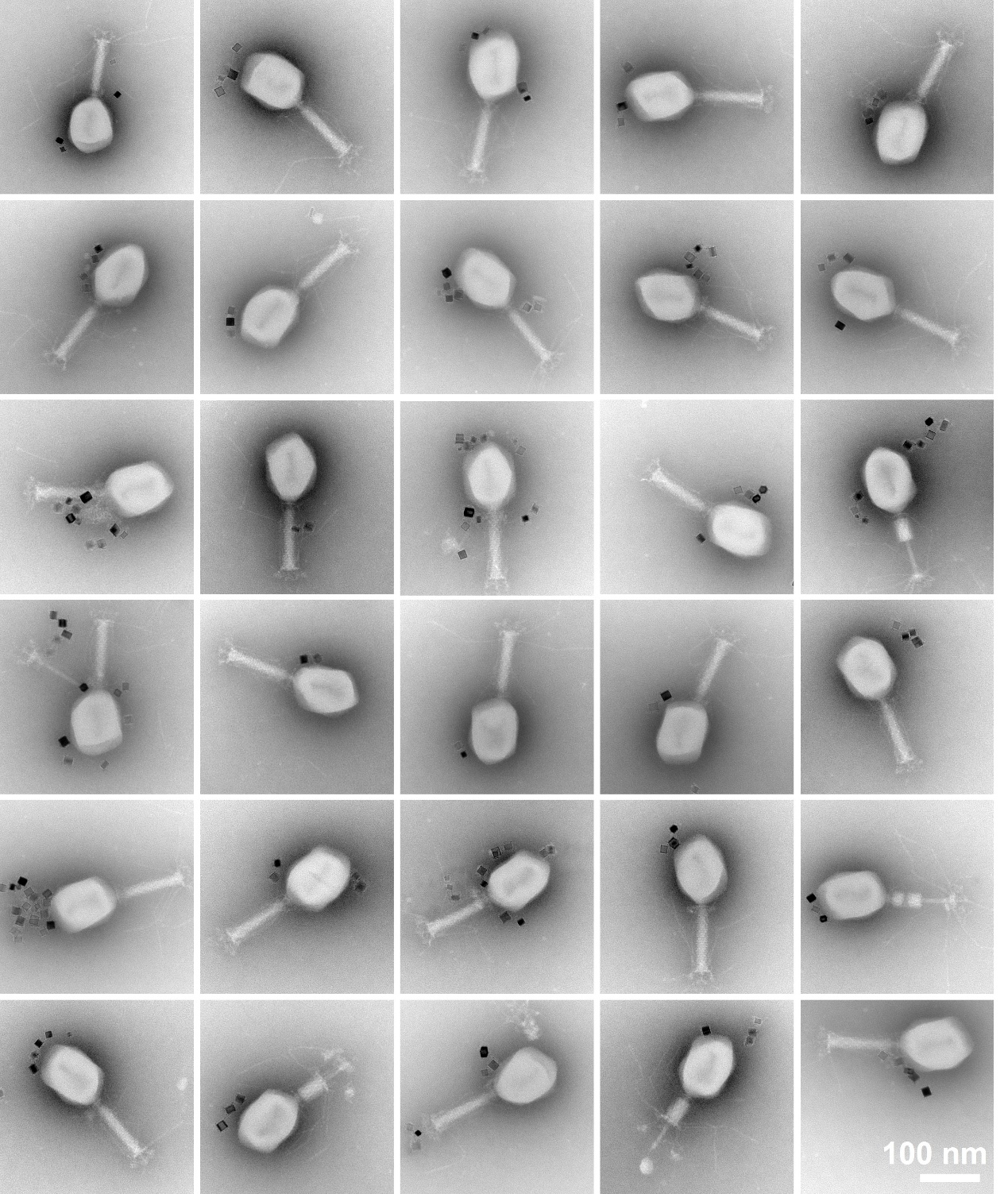
*

**Fig. S6** TEM images of multiple N4@Pd nanorobots showing consistent association of Pd nanozymes with bacteriophage N4. Scale bar = 100 nm.





**Fig. S7** Hydrodynamic size distributions of (A) Pd nanozymes (29.8 ± 6.2 nm), (B) phage N4 (83.6 ± 14.7 nm), and (C) N4@Pd nanorobots (96.4 ± 18.2 nm), measured by DLS.


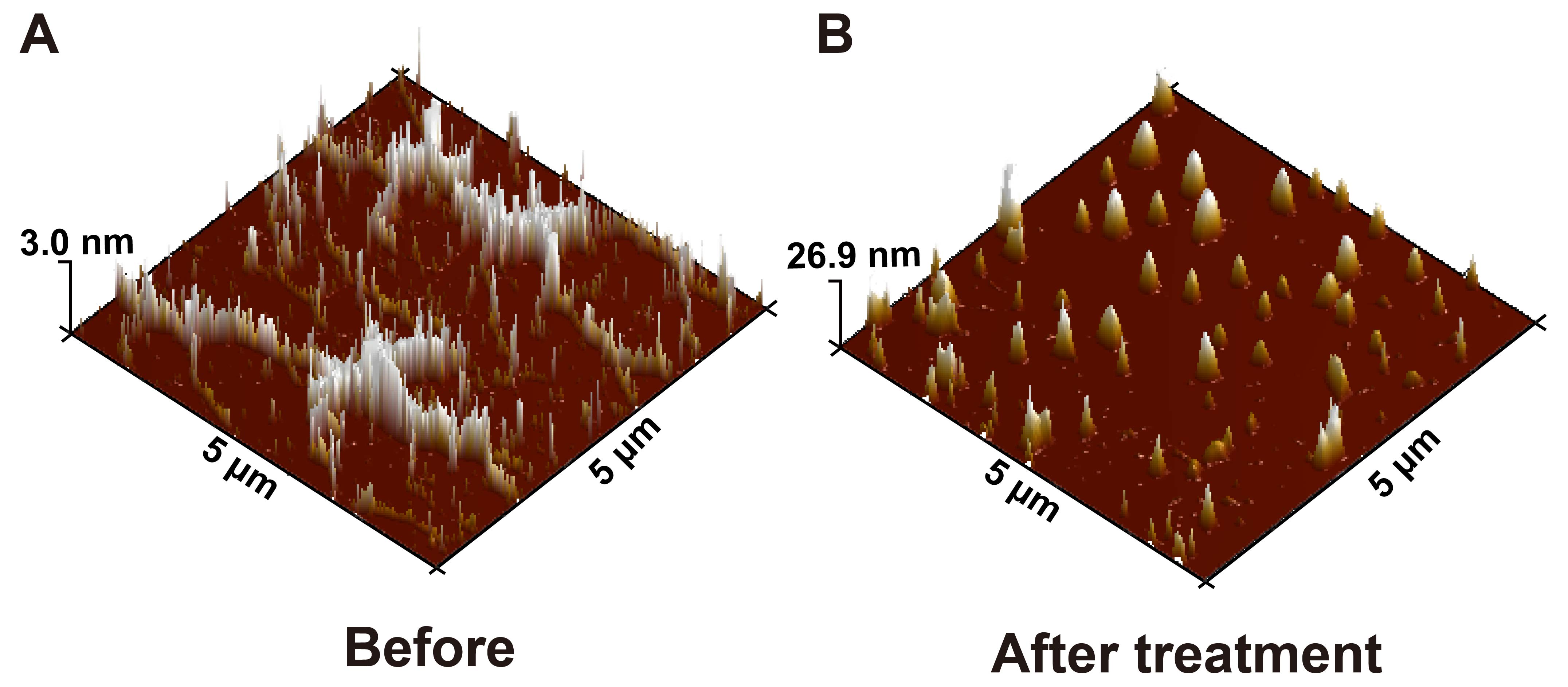


**Fig. S8** AFM 3D image of plasmid DNA (A) before and (B) after treatment with Pd (50 μg·mL^-1^ Pd) + H_2_O_2_ (1 mM) for 6 h, showing the treatment-induced morphological changes.


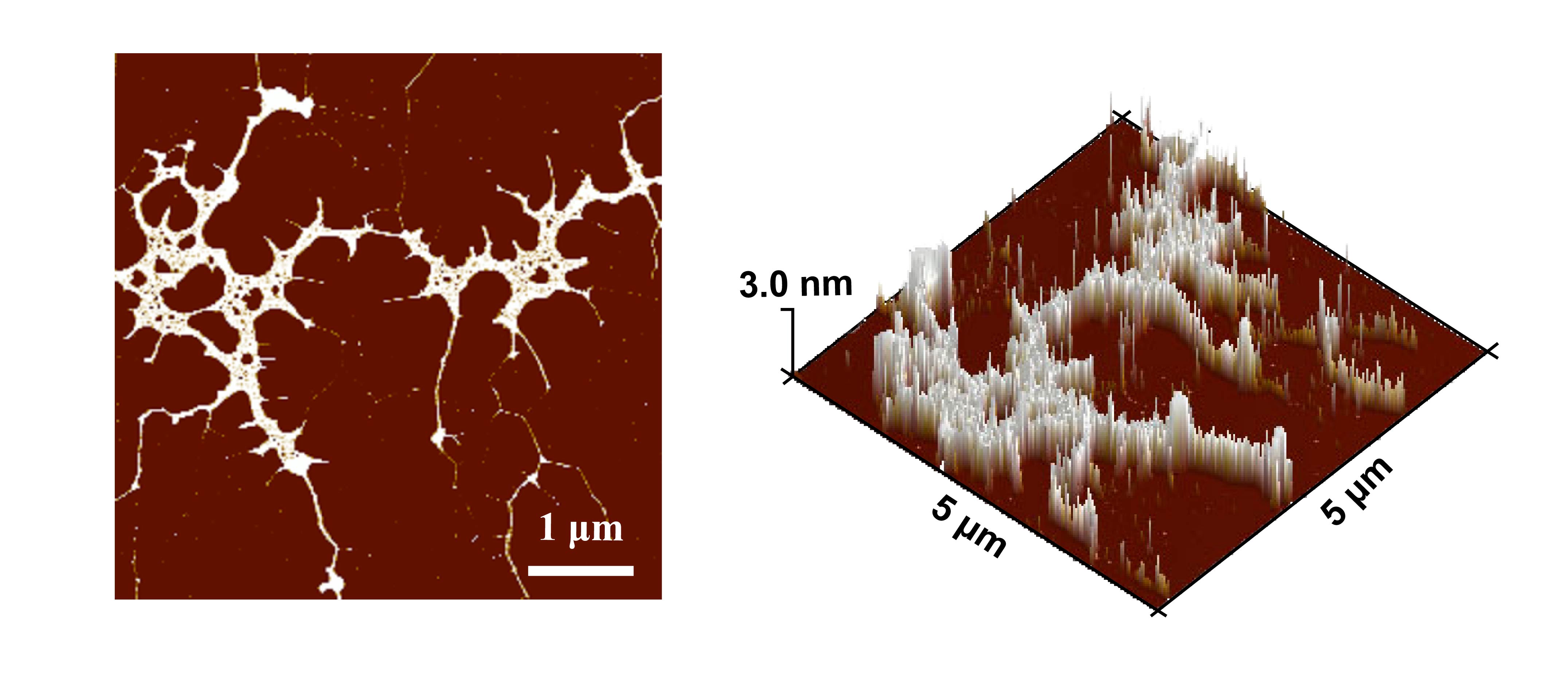


**Fig. S9** AFM height images and corresponding 3D topographic profiles of plasmid DNA treated with H_2_O_2_ (1 mM) alone for 6 h.

**
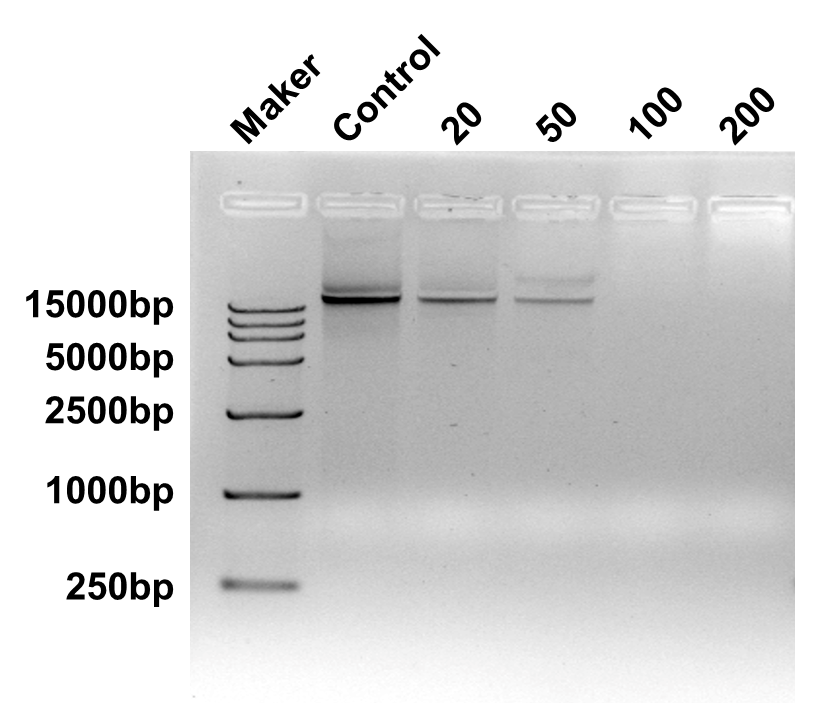
**

**Fig. S10** Agarose gel image of plasmid DNA degradation under different concentrations of Pd nanozyme (0-200 μg·mL^-1^) and H_2_O_2_ (1 mM) treatment for 6 h.

**
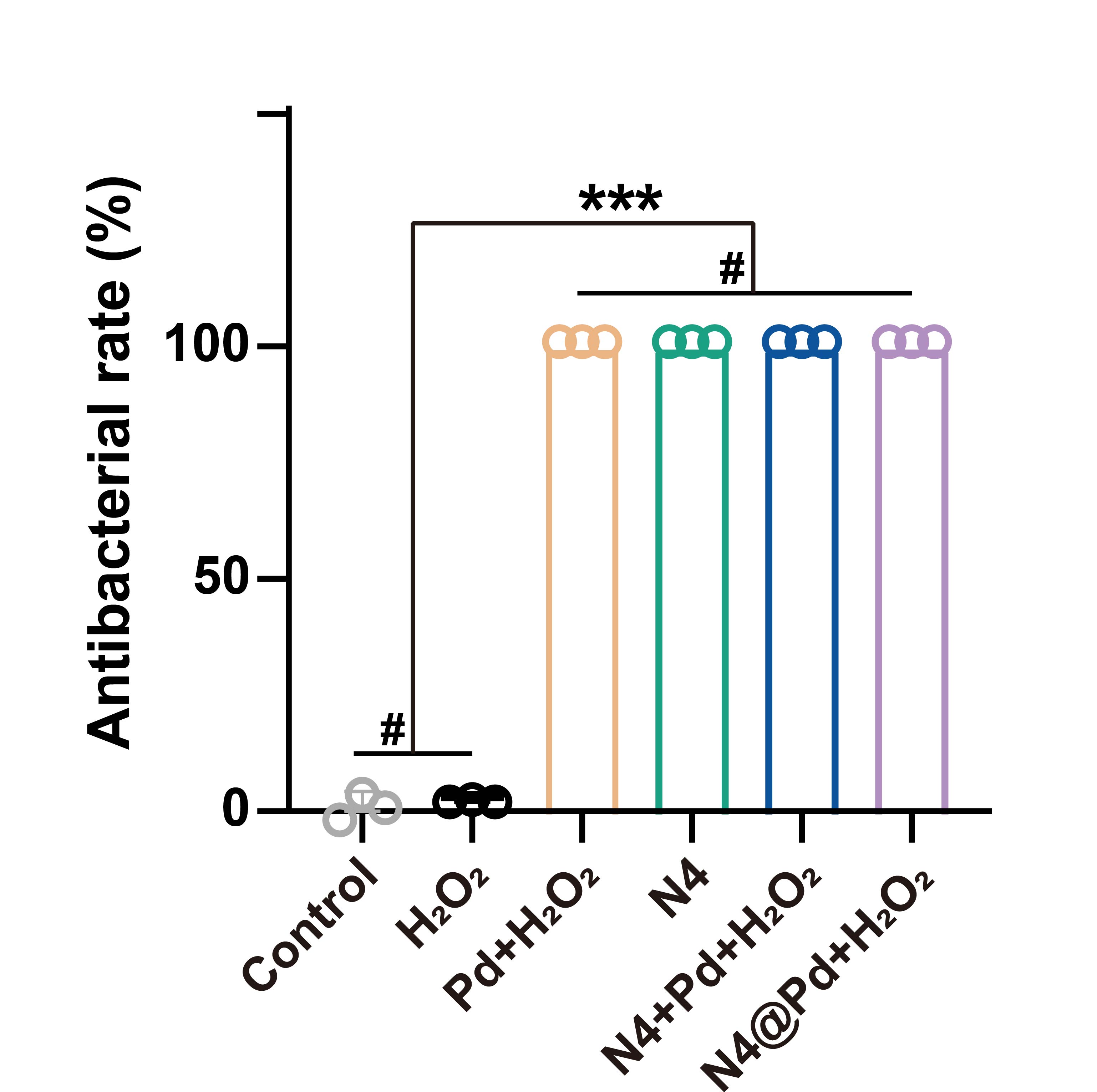
**

**Fig. S11** Antibacterial activity under different treatments (n = 3). *E. coli* NDM-1 was treated with PBS (control), H_2_O_2_ (1 mM), Pd (50 μg·mL^-1^) + H_2_O_2_ (1 mM), N4 (1×10^10^ PFU·mL^-1^), Pd + N4 + H_2_O_2_ (same concentrations as above), and N4@Pd (containing the corresponding N4 and Pd doses) + H_2_O_2_ (1 mM) in PBS (pH = 6) for 6 h. Data are presented as mean ± SD. Group differences were analyzed by one-way ANOVA followed by Tukey’s HSD post-hoc test. Significance was indicated as **P* < 0.05, ***P* < 0.01, ****P* < 0.001 and **#** indicates no significant difference (*P* ≥ 0.05).


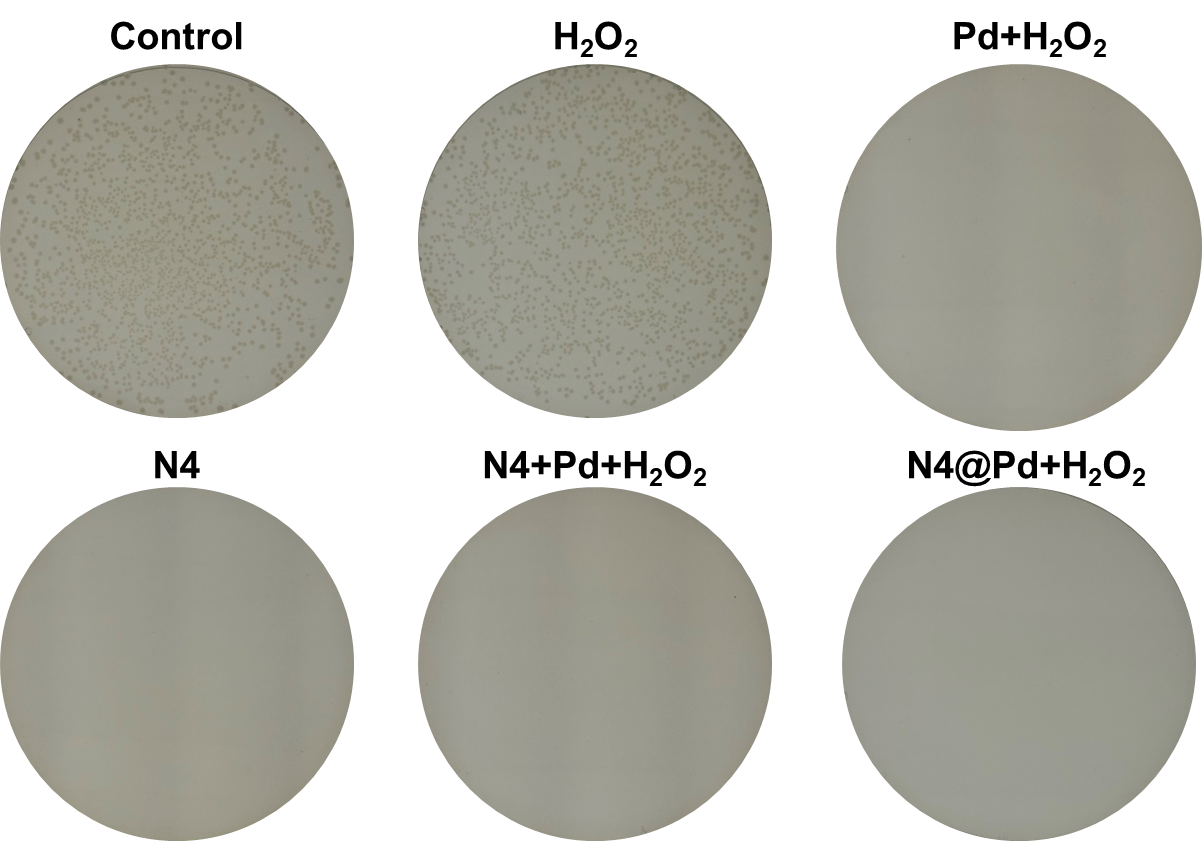


**Fig. S12** Images of bacterial colonies of *E. coli* NDM-1 on the culture plate after corresponding treatment. *E. coli* NDM-1 was treated with PBS (control), H_2_O_2_ (1 mM), Pd (50 μg·mL^-1^) + H_2_O_2_ (1 mM), N4 (1×10^10^ PFU·mL^-1^), Pd + N4 + H_2_O_2_ (same concentrations as above), and N4@Pd (containing the corresponding N4 and Pd doses) + H_2_O_2_ (1 mM) in PBS (pH = 6) for 6 h.

**
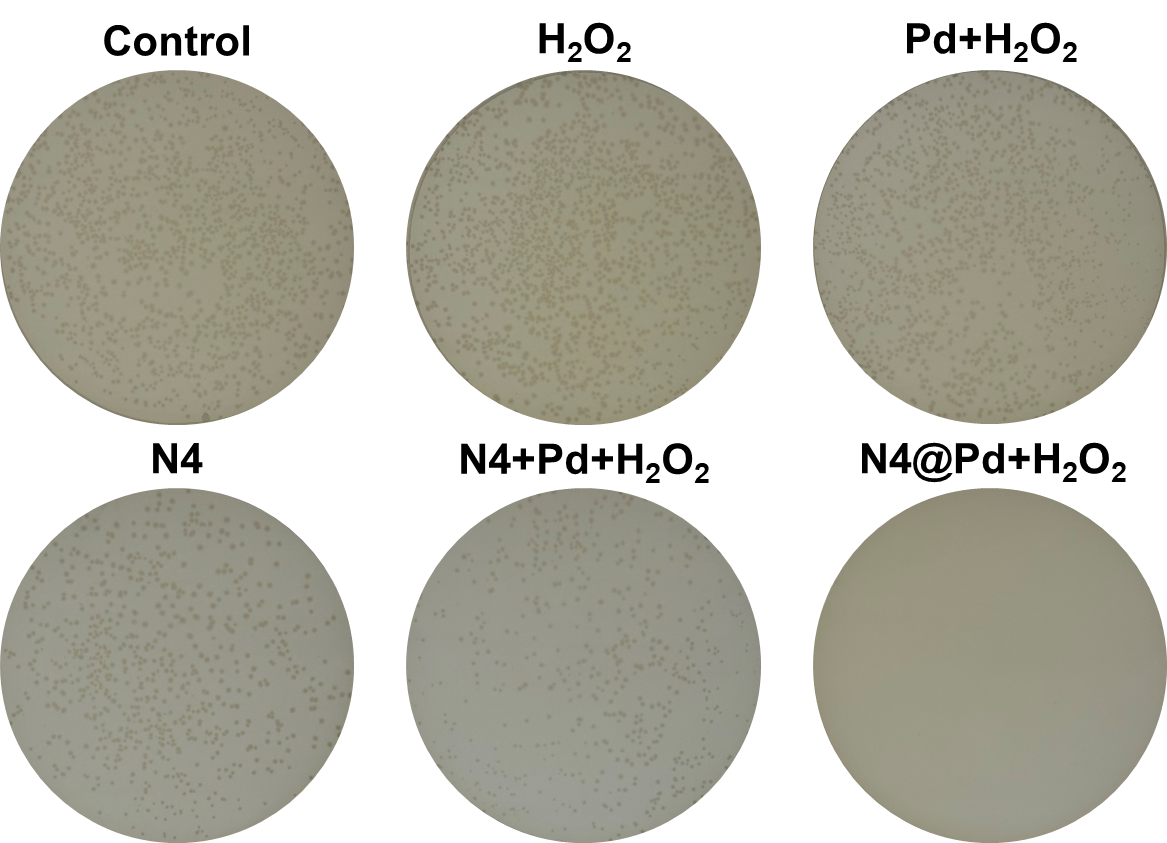
**

**Fig. S13** Images of bacterial colonies of *E. coli* NDM-1 on the culture plate after corresponding treatment (low concentration). *E. coli* NDM-1 was treated with PBS (control), H_2_O_2_ (1 mM), Pd (50 ng·mL^-1^) + H_2_O_2_ (1 mM), N4 (1×10^7^ PFU·mL^-1^), Pd + N4 + H_2_O_2_ (same concentrations as above), and N4@Pd (containing the corresponding N4 and Pd doses) + H_2_O_2_ (1 mM) in PBS (pH = 6) for 6 h.

**
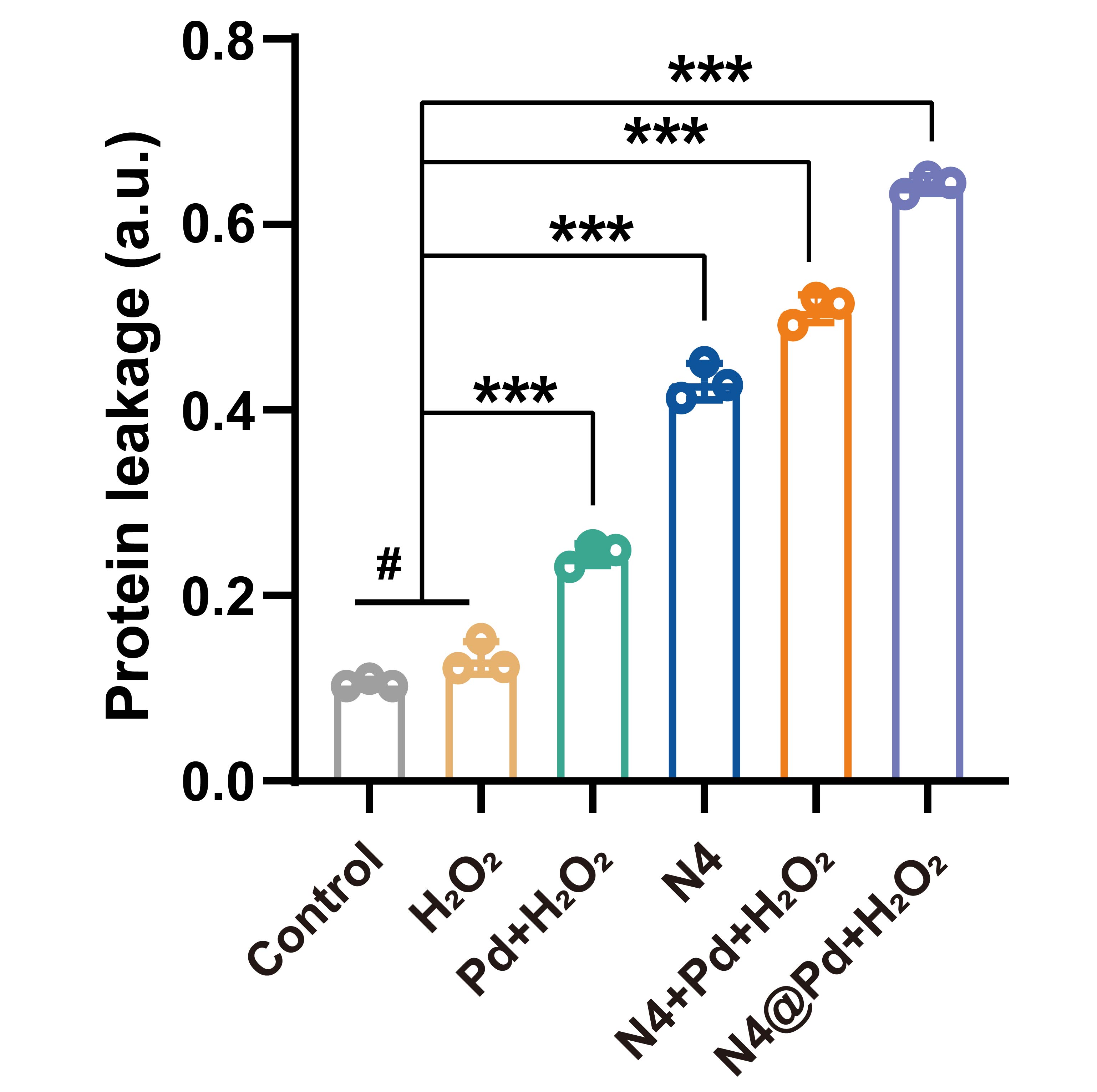
**

**Fig. S14** Protein leakage levels from *E. coli* NDM-1 caused by various treatments (n = 3). *E. coli* NDM-1 was treated with PBS (control), H_2_O_2_ (1 mM), Pd (50 ng·mL^-1^) + H_2_O_2_ (1 mM), N4 (1×10^10^ PFU·mL^-1^), Pd + N4 + H_2_O_2_ (same concentrations as above), and N4@Pd (containing the corresponding N4 and Pd doses) + H_2_O_2_ (1 mM) in PBS (pH = 6) for 6 h. Data are presented as mean ± SD. Group differences were analyzed by one-way ANOVA followed by Tukey’s HSD post-hoc test. Significance was indicated as **P* < 0.05, ***P* < 0.01, ****P* < 0.001 and **#** indicates no significant difference (*P* ≥ 0.05).

**
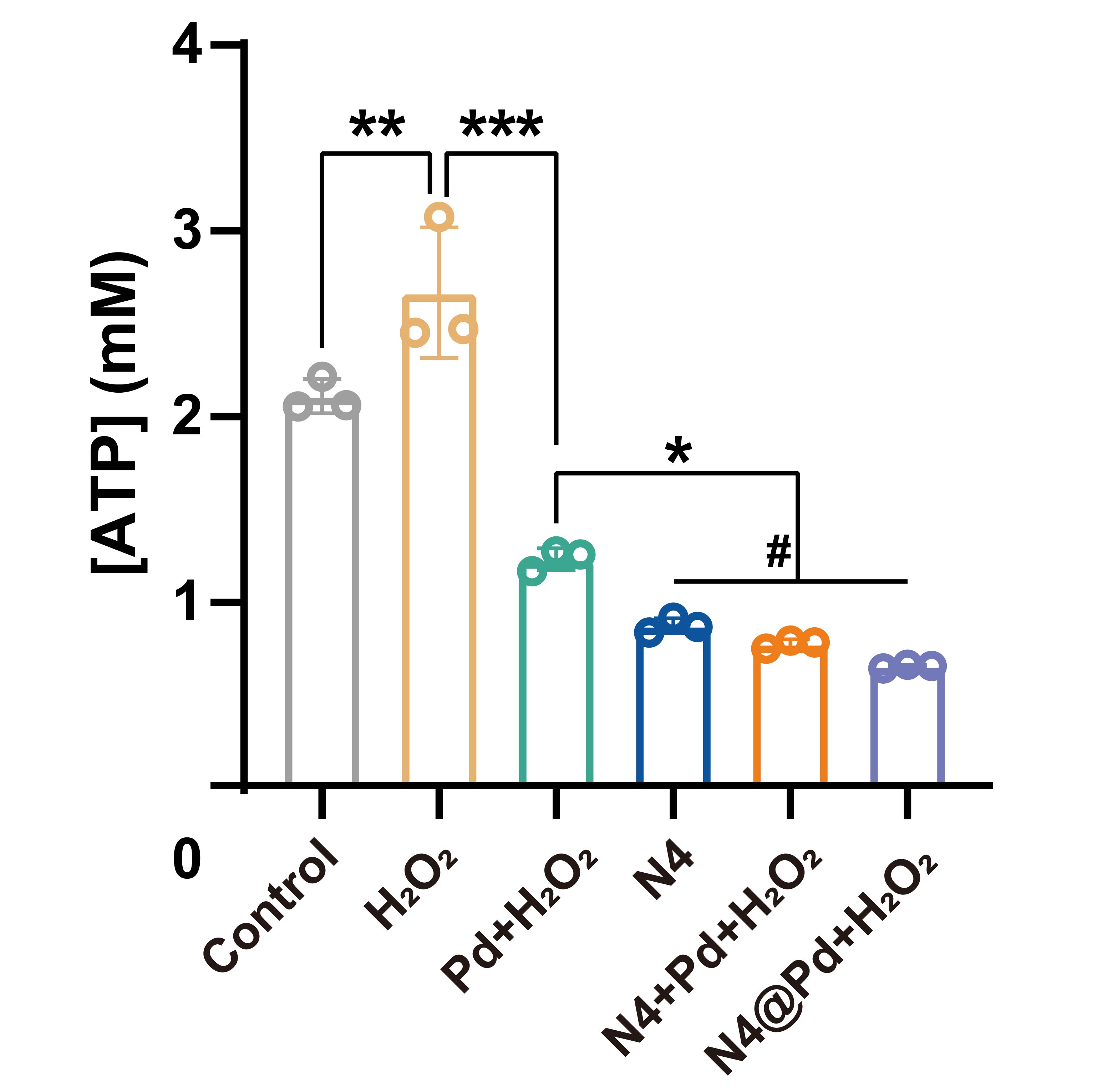
**

**Fig. S15** Intracellular ATP levels after different treatments (n = 3). *E. coli* NDM-1 was treated with PBS (control), H_2_O_2_ (1 mM), Pd (50 ng·mL^-1^) + H_2_O_2_ (1 mM), N4 (1×10^10^ PFU·mL^-1^), Pd + N4 + H_2_O_2_ (same concentrations as above), and N4@Pd (containing the corresponding N4 and Pd doses) + H_2_O_2_ (1 mM) in PBS (pH = 6) for 6 h. Data are presented as mean ± SD. Group differences were analyzed by one-way ANOVA followed by Tukey’s HSD post-hoc test. Significance was indicated as **P* < 0.05, ***P* < 0.01, ****P* < 0.001 and **#** indicates no significant difference (*P* ≥ 0.05).


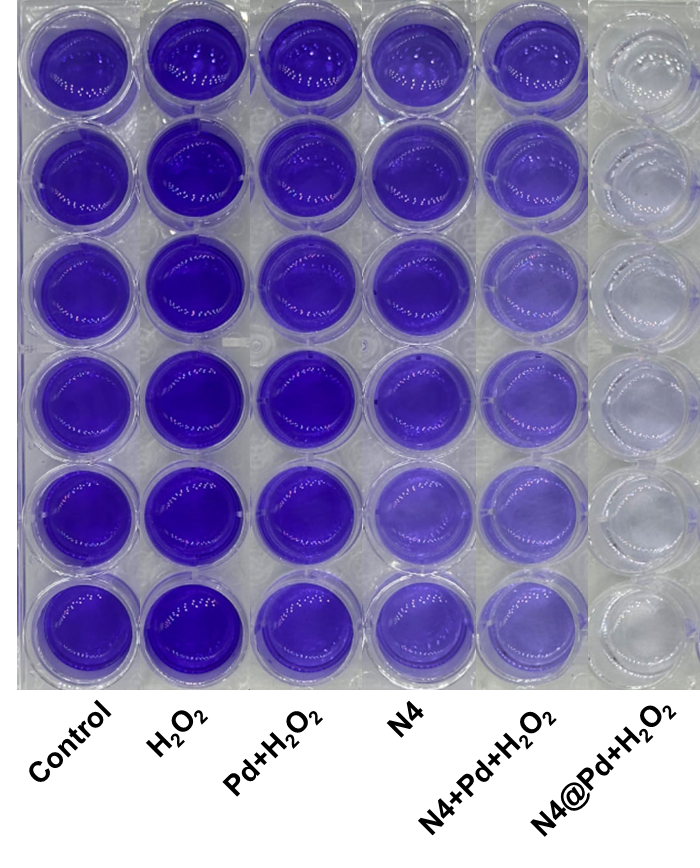


**Fig. S16** Qualitative analysis of the biomass of biofilms by crystal violet staining. Biofilm was treated with PBS (control), H_2_O_2_ (1 mM), Pd (50 μg·mL^-1^) + H_2_O_2_ (1 mM), N4 (1×10^10^ PFU·mL^-1^), Pd + N4 + H_2_O_2_ (same concentrations as above), and N4@Pd (containing the corresponding N4 and Pd doses) + H_2_O_2_ (1 mM) for 12 h (n = 6).


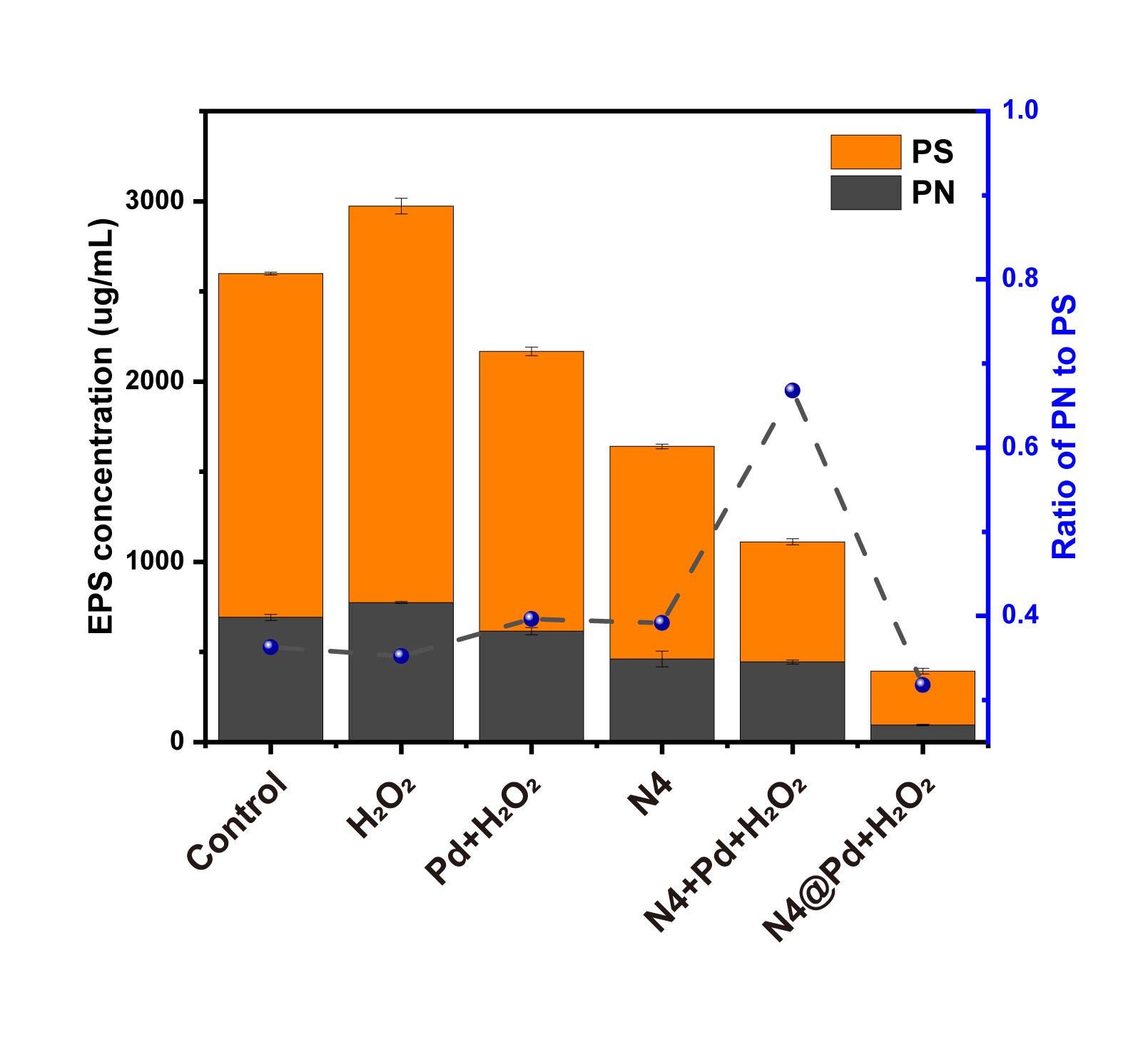


**Fig. S17** Protein and polysaccharide contents and ratio of PN to PS in *E. coli* NDM-1. Biofilm was treated with PBS (control), H_2_O_2_ (1 mM), Pd (50 μg·mL^-1^) + H_2_O_2_ (1 mM), N4 (1×10^10^ PFU·mL^-1^), Pd + N4 + H_2_O_2_ (same concentrations as above), and N4@Pd (containing the corresponding N4 and Pd doses) + H_2_O_2_ (1 mM) for 12 h (n = 6).


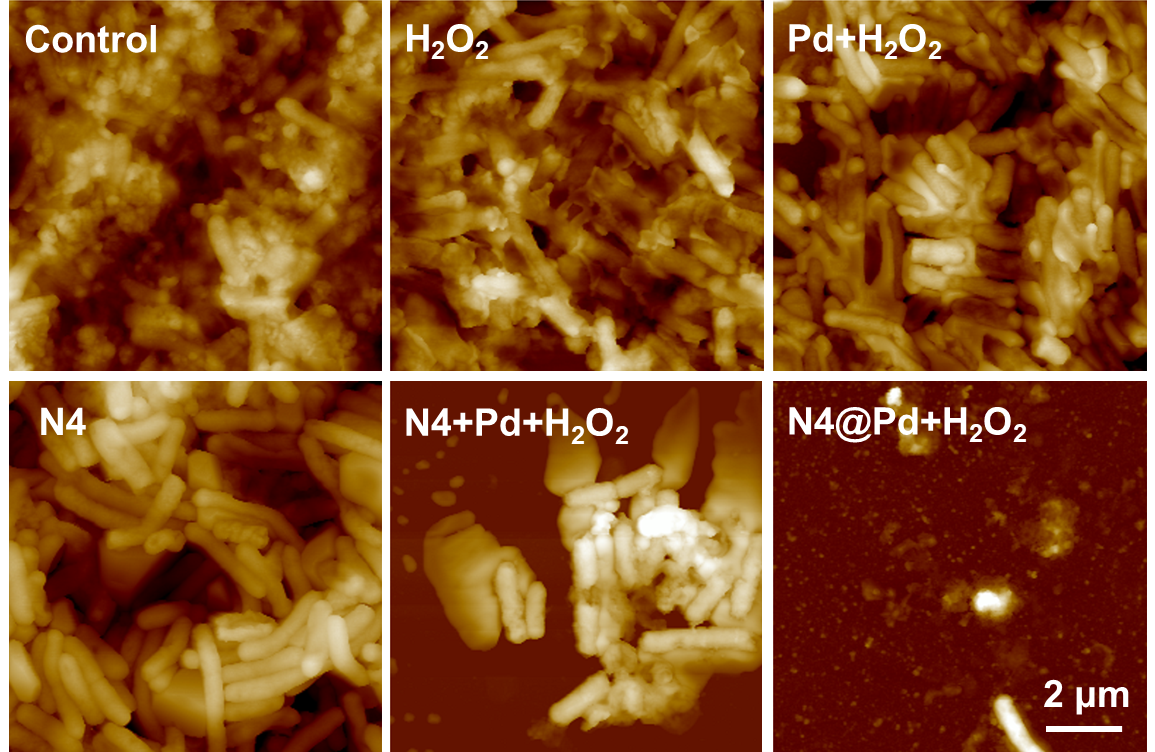


**Fig. S18** AFM images of *E. coli* NDM-1 biofilm after corresponding treatment. Scale bar = 2 μm. Biofilm was treated with PBS (control), H_2_O_2_ (1 mM), Pd (50 μg·mL^-1^) + H_2_O_2_ (1 mM), N4 (1×10^10^ PFU·mL^-1^), Pd + N4 + H_2_O_2_ (same concentrations as above), and N4@Pd (containing the corresponding N4 and Pd doses) + H_2_O_2_ (1 mM) for 12 h (n = 6).


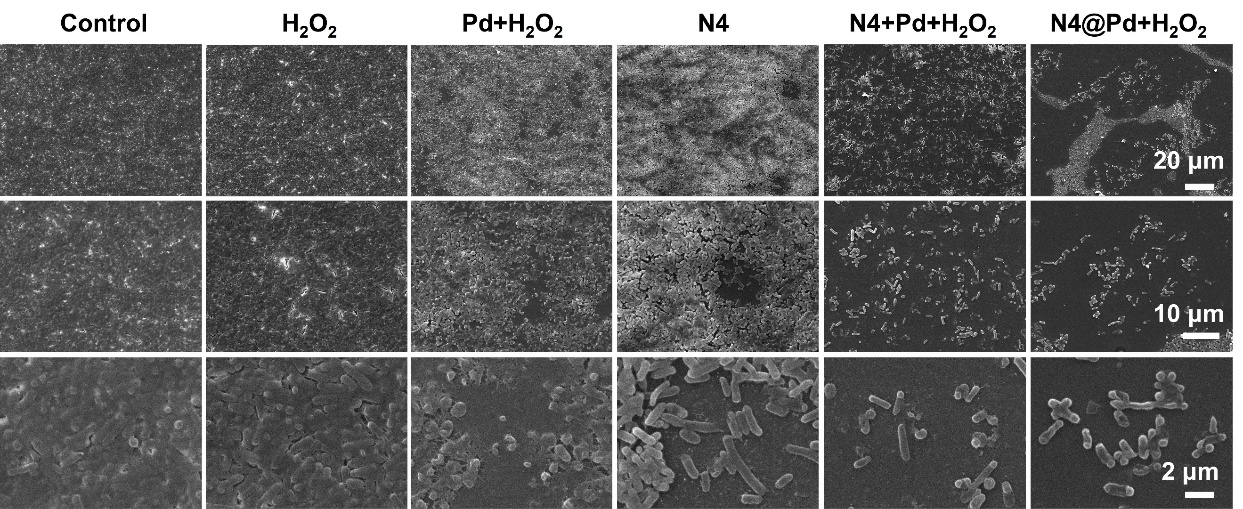


**Fig. S19** SEM images of *E. coli* NDM-1 biofilm after corresponding treatment. Scale bar = 20, 10, and 2 μm. Biofilm was treated with PBS (control), H_2_O_2_ (1 mM), Pd (50 μg·mL^-1^) + H_2_O_2_ (1 mM), N4 (1×10^10^ PFU·mL^-1^), Pd + N4 + H_2_O_2_ (same concentrations as above), and N4@Pd (containing the corresponding N4 and Pd doses) + H_2_O_2_ (1 mM) for 12 h (n = 6).

**
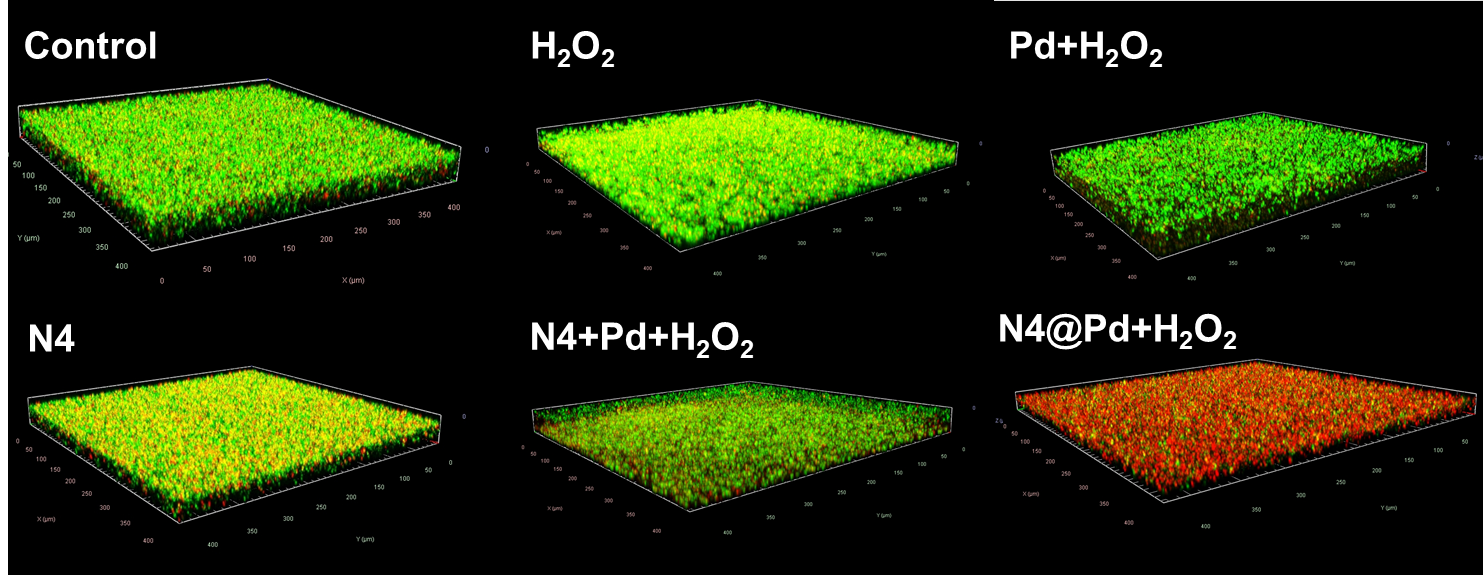
**

**Fig. S20** 3D CLSM images of *E. coli* NDM-1 biofilm after corresponding treatment. Biofilm was treated with PBS (control), H_2_O_2_ (1 mM), Pd (50 μg·mL^-1^) + H_2_O_2_ (1 mM), N4 (1×10^10^ PFU·mL^-1^), Pd + N4 + H_2_O_2_ (same concentrations as above), and N4@Pd (containing the corresponding N4 and Pd doses) + H_2_O_2_ (1 mM) for 12 h (n = 6).


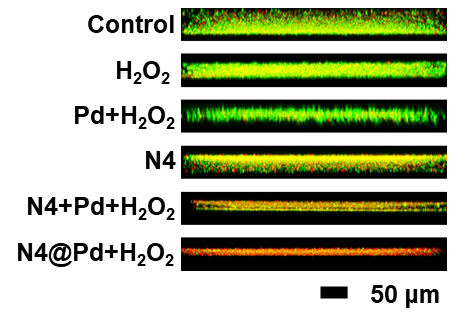


**Fig. S21** CLSM image of the thickness changes of *E. coli* NDM-1 biofilm after corresponding treatment. Scale bar = 50 μm. Biofilm was treated with PBS (control), H_2_O_2_ (1 mM), Pd (50 μg·mL^-1^) + H_2_O_2_ (1 mM), N4 (1×10^10^ PFU·mL^-1^), Pd + N4 + H_2_O_2_ (same concentrations as above), and N4@Pd (containing the corresponding N4 and Pd doses) + H_2_O_2_ (1 mM) for 12 h (n = 6).

**
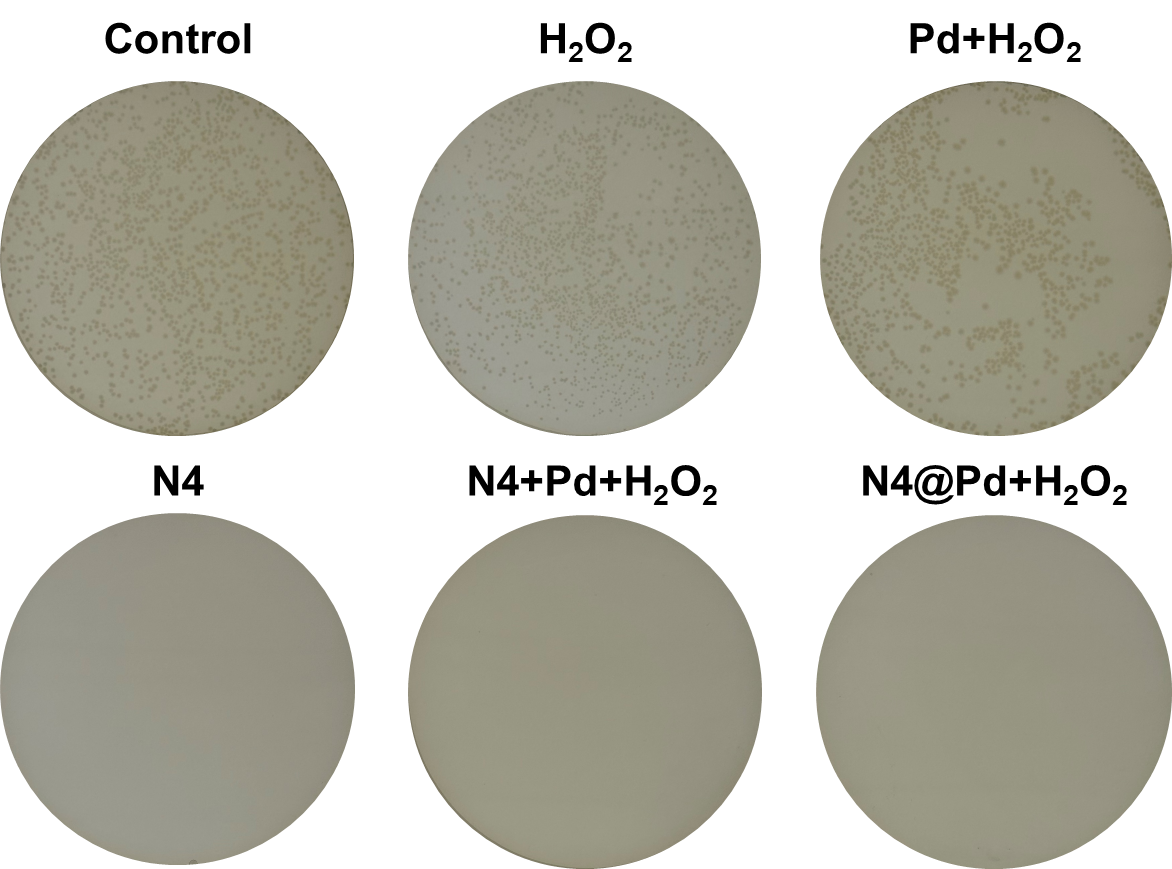
**

**Fig. S22** Images of bacterial colonies of *E. coli* NDM-1 on the culture plate after corresponding treatment in simulated wastewater. *E. coli* NDM-1 was treated with PBS (control), H_2_O_2_ (1 mM), Pd (50 μg·mL^-1^) + H_2_O_2_ (1 mM), N4 (1×10^10^ PFU·mL^-1^), Pd + N4 + H_2_O_2_ (same concentrations as above), and N4@Pd (containing the corresponding N4 and Pd doses) + H_2_O_2_ (1 mM) in simulated wastewater for 6 h (n = 3).


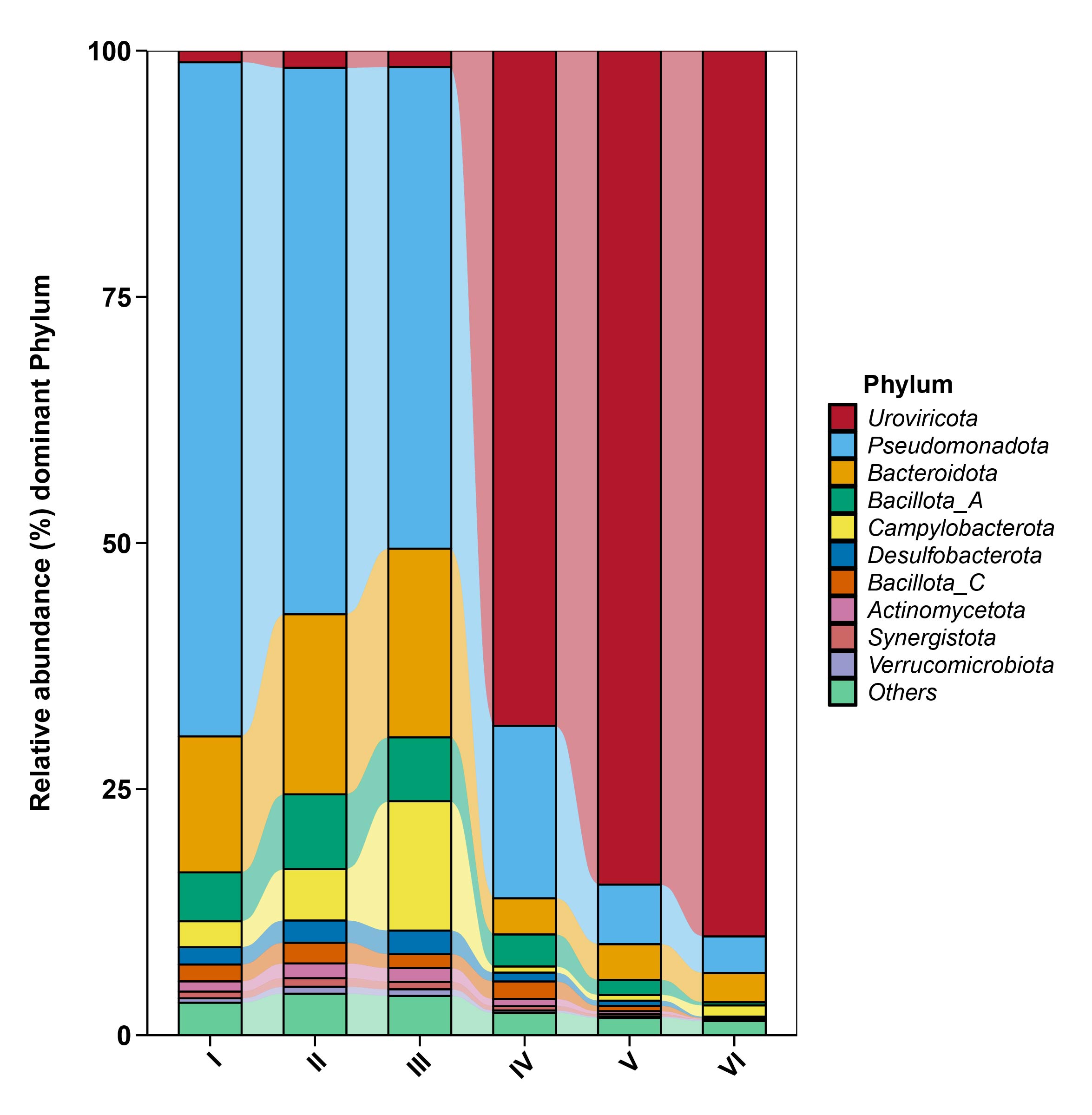


**Fig. S23** Relative abundance of dominant bacterial genera in each treatment group. Real wastewater inoculated with E. coli NDM-1 was treated for 6 h with PBS (control), H_2_O_2_ (1 mM), Pd (50 μg·mL^-1^) + H_2_O_2_ (1 mM), N4 (1×10^10^ PFU·mL^-1^), Pd + N4 + H_2_O_2_ (same concentrations as above), and N4@Pd (containing the corresponding N4 and Pd doses) + H_2_O_2_ (1 mM) (n = 3).


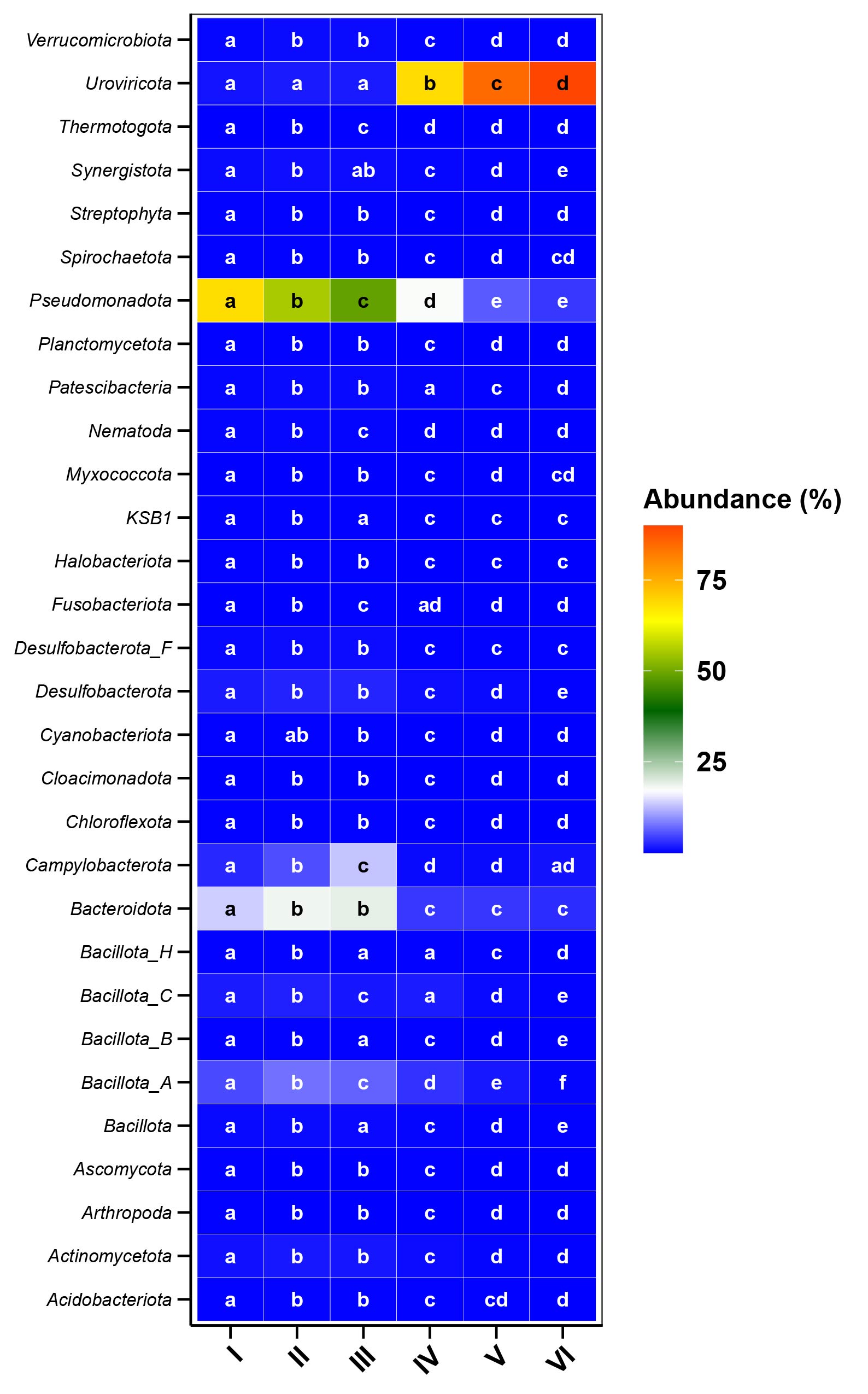


**Fig. S24** Heatmap showing the differential abundance of species across different groups. Real wastewater inoculated with E. coli NDM-1 was treated for 6 h with PBS (control), H_2_O_2_ (1 mM), Pd (50 μg·mL^-1^) + H_2_O_2_ (1 mM), N4 (1×10^10^ PFU·mL^-1^), Pd + N4 + H_2_O_2_ (same concentrations as above), and N4@Pd (containing the corresponding N4 and Pd doses) + H_2_O_2_ (1 mM) (n = 3).


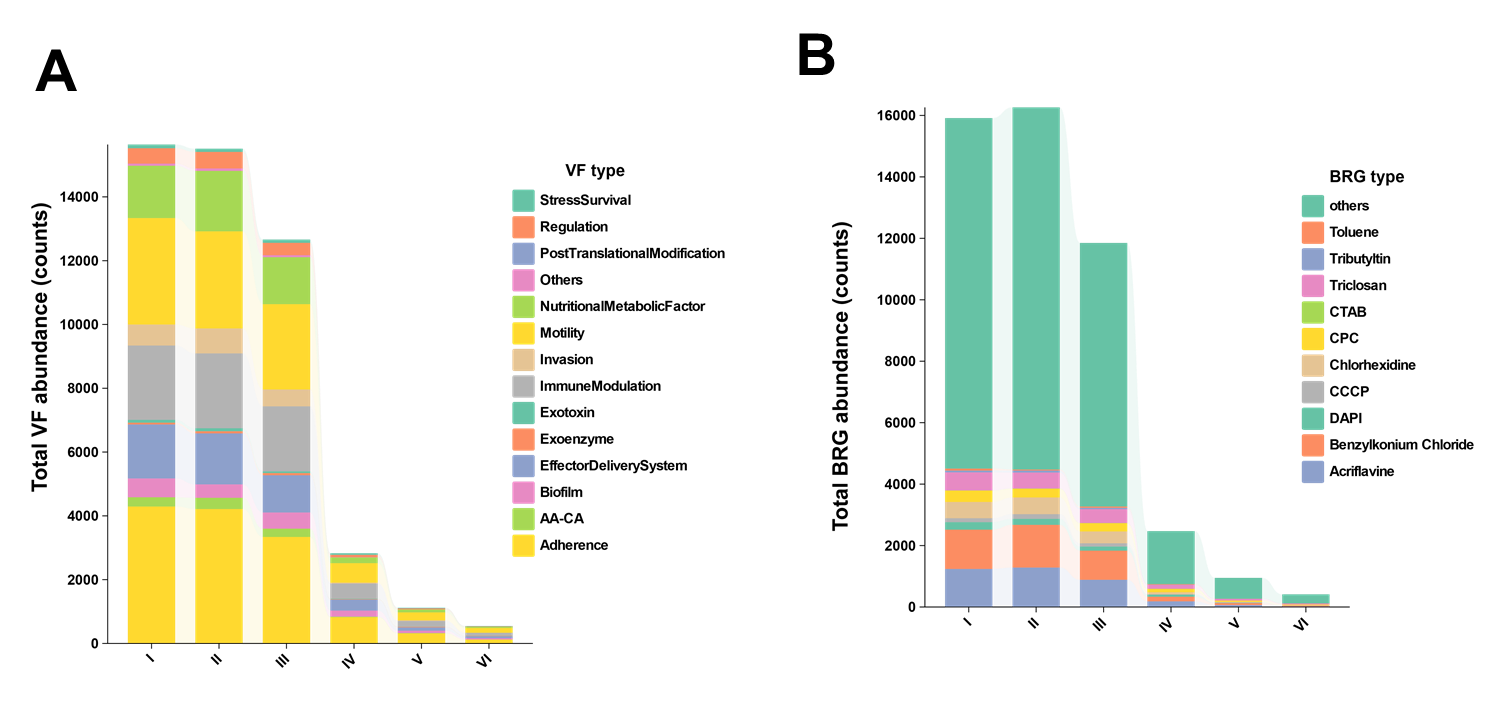


**Fig. S25** Total abundance of biocide resistance genes (BRG) across different groups. Real wastewater inoculated with E. coli NDM-1 was treated for 6 h with PBS (control), H_2_O_2_ (1 mM), Pd (50 μg·mL^-1^) + H_2_O_2_ (1 mM), N4 (1×10^10^ PFU·mL^-1^), Pd + N4 + H_2_O_2_ (same concentrations as above), and N4@Pd (containing the corresponding N4 and Pd doses) + H_2_O_2_ (1 mM) (n = 3).

**Table S1 Composition of artificially simulated wastewater**

| **Main nutritional components** | **Concentration**  **(mg/L)** | **Trace element** | **Concentration**  **(mg/L)** |
| --- | --- | --- | --- |
| CH_3_COONa | 600 | H_3_BO_3_ | 0.15 |
| NH_4_Cl | 150 | MnCl_2_·4H_2_O | 0.12 |
| KH_2_PO_4_ | 30 | CuSO_4_·5H_2_O | 0.03 |
| MgSO_4_·7H_2_O | 50 | ZnSO_4_·7H_2_O | 0.12 |
| CaCl_2_ | 10 | Na_2_MoO_4_·2H_2_O | 0.06 |
| FeSO_4_·7H_2_O | 10 | KI | 0.18 |
| Humic acid | 10 | CoCl_2_·6H_2_O | 0.15 |

# 3. References

[1] C. Mao, Y. Xiang, X. Liu, Y. Zheng, K. W. K. Yeung, Z. Cui, X. Yang, Z. Li, Y. Liang, S. Zhu, S. Wu, *ACS Appl. Mater. Interfaces* **2019**, *11* (19), 17902, https://doi.org/10.1021/acsami.9b05787.

[2] R. Long, K. Mao, X. Ye, W. Yan, Y. Huang, J. Wang, Y. Fu, X. Wang, X. Wu, Y. Xie, Y. Xiong, *J. Am. Chem. Soc.* **2013**, *135* (8), 3200, https://doi.org/10.1021/ja311739v.

[3] Y. Zhang, S. Zuo, Q. Zheng, G. Yu, Y. Wang, *Water Res.* **2024**, *265*, 122298, https://doi.org/10.1016/j.watres.2024.122298.

[4] Y. Fu, F. Wang, H. Sheng, F. Hu, Z. Wang, M. Xu, Y. Bian, X. Jiang, J. M. Tiedje, *J. Hazard. Mater.* **2021**, *411*, 125048, https://doi.org/10.1016/j.jhazmat.2021.125048.

[5] D. Li, P. Yu, X. Zhou, J.-H. Kim, Y. Zhang, P. J. J. Alvarez, *Water Res.* **2020**, *184*, 116157, https://doi.org/10.1016/j.watres.2020.116157.
